# Inflammatory Factor IL1α Induces Aberrant Astrocyte Proliferation in Spinal Cord Injury Through the Grin2c/Ca^2+^/CaMK2b Pathway

**DOI:** 10.1007/s12264-023-01128-4

**Published:** 2023-10-21

**Authors:** Yu Xia, Lu Ding, Changlin Zhang, Qi Xu, Ming Shi, Tianshun Gao, Feng-Quan Zhou, David Y. B. Deng

**Affiliations:** 1 Scientific Research Center, The Seventh Affiliated Hospital of Sun Yat-sen University, Shenzhen, 518107 China; 2 Department of Gynecology, The Seventh Affiliated Hospital of Sun Yat-sen University, Shenzhen, 518107 China; 3 Pelvic Floor Disorders Center, The Seventh Affiliated Hospital of Sun Yat-sen University, Shenzhen, 518107 China; 4 Big Data Center, The Seventh Affiliated Hospital of Sun Yat-sen University, Shenzhen, 518107 China; 5 Sir Run Run Shaw Hospital, Zhejiang University School of Medicine, Hangzhou, 310016 China; 6 Orthopaedic and Neurological Repair Center, The Seventh Affiliated Hospital of Sun Yat-sen University, Shenzhen, 518107 China

**Keywords:** IL1α, Grin2c, Astrocyte, Spinal cord injury

## Abstract

Spinal cord injury (SCI) is one of the most devastating traumas, and the aberrant proliferation of astrocytes usually causes neurological deficits. However, the mechanism underlying astrocyte over-proliferation after SCI is unclear. Grin2c (glutamate ionotropic receptor type 2c) plays an essential role in cell proliferation. Our bioinformatic analysis indicated that Grin2c and Ca^2+^ transport functions were inhibited in astrocytes after SCI. Suppression of Grin2c stimulated astrocyte proliferation by inhibiting the Ca^2+^/calmodulin-dependent protein kinase 2b (CaMK2b) pathway *in vitro*. By screening different inflammatory factors, interleukin 1α (IL1α) was further found to inhibit Grin2c/Ca^2+^/CaMK2b and enhance astrocyte proliferation in an oxidative damage model. Blockade of IL1α using neutralizing antibody resulted in increased Grin2c expression and the inhibition of astrocyte proliferation post-SCI. Overall, this study suggests that IL1α promotes astrocyte proliferation by suppressing the Grin2c/Ca^2+^/CaMK2b pathway after SCI, revealing a novel pathological mechanism of astrocyte proliferation, and may provide potential targets for SCI repair.

**Supplementary Information:**

The online version contains supplementary material available at 10.1007/s12264-023-01128-4.

## Introduction

Spinal cord injury (SCI) is a devastating injury caused by trauma to the spinal column [1], usually resulting in sensorimotor and autonomic nerve damage [2]. Initial mechanical trauma to the spinal cord initiates a secondary injury cascade, including immune inflammation, neuroexcitatory toxicity, and accumulation of reactive oxygen species (ROS) and calcium ions (Ca^2+^), which further exacerbate the damage [3]. As the most abundant cell type in the central nervous system (CNS) [4], astrocytes are activated, then proliferate after injury and response to microenvironmental factors, such as inflammatory factors (IL1α, C1q, and TNFα) [5]. In particular, the abnormal proliferation of astrocytes contributes to the formation of glial scars [6], which cause a major barrier to functional recovery by displacing nerve structure and preventing axon regeneration [7]. The aberrant activation of astrocytes can also participate in secondary injury by mediation of excitatory neurotoxicity, tissue edema, and blood-brain barrier breakdown [8]. Therefore, aberrant astrocyte proliferation plays an important role in the process of SCI and the underlying mechanism deserves further study and exploration.

The change in intracellular Ca^2+^ levels is a major area of interest in the mechanistic study of the abnormal activation of astrocytes [9]. The intracellular Ca^2+^ level usually increases after CNS injury and induces cell death through a variety of pathways, such as apoptosis, pyroptosis, and the accumulation of free radicals [10, 11]. However, the intracellular Ca^2+^ level in astrocytes declines after the initial increase post-SCI [12]. Trauma to CNS can strengthen Ca^2+^ regulation in the proliferative astrocytes, which have an enhanced ability to withstand supraphysiological Ca^2+^ loads. Alteration of Ca^2+^ levels is associated with reactive astrogliosis [13] and causes changes in self-modulating proliferation, morphology, movement, phagocytosis, and material balance in astrocytes [14, 15]. Ca^2+^ concentrations are normally regulated by absorption and release from intracellular mitochondria and endoplasmic reticulum [16]. In addition, the alteration of intracellular Ca^2+^ levels is also dependent on the osmotic role of Ca^2+^ *via* the Ca^2+^ ionophore of the cellular membrane [17].

The N-methyl-D-aspartate receptor (NMDAR) is an ionotropic glutamate receptor that is Ca^2+^ permeable, which allows Ca^2+^ to continuously enter the cell [18] and activates calmodulin-dependent protein kinase (CaMK) to modulate cellular states [19]. For example, the changes in the current ratio and the increased number of silent synapses induced by the NADMR function as a potential mechanism of neurodegeneration occurring in Alzheimer's disease [20]. In contrast to other NMDAR subunits localized in neurons, Grin2c is highly expressed in astrocytes [21, 22]. Notably, Grin2c may act as one of the disease-associated variants to regulate cell proliferation and death [23, 24]. For instance, Grin2c can activate the phosphorylation cycle of cyclic adenosine monophosphate response element-binding protein to regulate cellular activity through the activation of CaMK2b in shift work sleep disorder [25].

In the present study, to explore the mechanism of aberrant astrocyte proliferation, we applied bioinformatic analysis of high-throughput sequencing data from astrocytes after SCI in the GEO database (GSE153720), and the results revealed that Grin2c may be involved in astrocyte proliferation by downregulating Ca^2+^ pathways. The expression of Grin2c in astrocytes was significantly downregulated in SCI model mice. The downregulation of Grin2c by siRNA *in vitro* enhanced astrocyte proliferation by inhibiting Ca^2+^/CaMK2b signaling. Furthermore, using inflammatory factor screening, we further found that IL1α inhibited Grin2c and the downstream Ca^2+^/CaMK2b pathway to facilitate astrocyte proliferation in an *in vitro* model of oxidative damage. Blockade of IL1α in SCI model mice using a neutralizing antibody reversed the decrease in Grin2c levels in astrocytes and suppressed the proliferation of astrocytes post-SCI, further leading to a reduction in lesion scar and lesion volume. This study revealed that Grin2c mediates aberrant astrocyte proliferation by inhibiting the Ca^2+^/CaMK2b pathway after SCI, which may offer new insights into treatments that promote nerve regeneration and SCI repair by reducing glial scar formation.

## Materials and Methods

### Bioinformatic Analysis

The Gene Expression Omnibus (GEO) dataset (GSE153720), which consists of expression profiling data of purified astrocytes after murine spinal cord injury generated by high throughput sequencing, was obtained from the GEO database (http://www.ncbi.nlm.nih.gov/geo/GSE153720). The R language limma (linear model for microarray analysis) package was used to perform differential gene expression analyses between SCI day 7 (the 7^th^ day after SCI) and Sham (sham operation) mice (inclusion criteria: abs(log_2_FC) >1, *P*-value <0.05). The *P*-value was calculated using a *t*-test statistic from the linear model [26]. Our Gene Ontology (GO) and Kyoto Encyclopedia of Genes and Genomes (KEGG) enrichment analyses were performed with the clusterProfiler and GSEA packages (v3.10.1). Here, we present these results through volcano plots, bubble plots, bar graphs, and heat maps. The composition of pathways was visualized using the analysis function in the R package Pathview (v 1.24.0). The same approach was applied to analyze the high-throughput sequencing of IL1α-stimulated astrocytes.

In a subsequent analysis and presentation of bioinformatic results, we processed data on several websites. The receptor-related gene set was downloaded from the GO website (http://geneontology.org/). The Venn diagram was generated using http://www.bioinformatics.com.cn. A volcano plot was generated using the web tools provided at http://www.bioinformatics.com.cn. Our analysis of interactions involved the STRING (https://string-db.org) functional protein association network. A database of Grin2c-related genes was obtained from GeneMania (http://genemania.org/).

### Animals and Ethics Approval

Adult C57BL/6 mice (half male and half female; age, 6−8 weeks; weight, 18−22 g) and newborn C57BL/6 mice (age, 1–2 days) were purchased from the Zhuhai Bestest Biotechnology Co. All animals used in the experiments were housed in the standard cages under standard specific pathogen-free conditions, with a regulated environment (12 h light/dark cycle) with free access to food and water for ~1 week before surgery. According to the Chinese national guidelines for animal experiments, all animal experiments were conducted strictly in accordance with the Regulations for the Administration of Affairs Concerning Experimental Animals.

All laboratory animal procedures were approved by the Animal Care and Use Committee (TOP-IACUC-2022-0127). The study was performed according to international, national, and institutional rules considering animal experiments and biodiversity rights. The study protocol was approved by the Ethics Committee of Sun Yat-sen University.

### Isolation and Culture of Mouse Astrocytes

C57BL/6 mice were sacrificed on days 1–2 to obtain cerebral astrocytes. Mice were anesthetized by CO_2_ narcosis and killed by cervical dislocation. A series of sterile surgical operations were performed, the skull was incised and the cerebral cortex was removed and stripped of the meninges and blood vessels. The tissue was washed twice with phosphate-buffer saline (Corning, NY, USA, 21-040-CV) and shredded with ophthalmic scissors. The minced tissue was then incubated for 5 min and filtered through a sieve with a 40 μm mesh size. After centrifugation at 1,000 r/min for 30 min, the mixed cells were cultured in Dulbecco’s modified Eagle’s medium (DMEM) (Corning, NY, USA, 10-0130-CVRC) with 1% antibiotic (Pricella, Wuhan, China, PB180120), and the medium was changed every 24–48 h. Seven days later, astrocytes were isolated by shaking the cells for 30 min, aspirating the supernatant, and changing the complete medium (DMEM + 10% fetal bovine serum (FBS) (BI, Kibbutz Beit Haemek, Israel, 04-001-1ACS) +1% antibiotic). The adhering cells were primary astrocytes. Passage of the cells was performed by trypsinization at 70–90% confluency, and plates of cells were prepared in advance according to experimental requirements.

### Construction of *In Vitro* Models to Simulate the SCI Environment

We used the following models to simulate the complicated environment of SCI *in vitro* (Fig. S5). To simulate the hypoxic-ischemic condition associated with SCI, oxygen-glucose deprivation (OGD) was used [27]. Through a tri-gas incubator (Panasonic, Osaka, Japan, MCO-18M), 95% N_2_ and 5% CO_2_ mixed gases were delivered to the chamber for 4 h, and then DMEM without FBS and glucose was added (Gibco, Grand Island, NY, USA, 11,966-025). Inflammatory models induced by gram-negative bacteria have been extensively validated [28]. Primary astrocytes were stimulated for 24 h with 100 ng/mL lipopolysaccharide (Beyotime, Shanghai, China, ST1470). To simulate the environment with the generation of ROS following an SCI, an oxidative damage model based on hydrogen peroxide (H_2_O_2_) (Sigma, St. Louis, MO, USA, 31642) was used [29]. A model of oxidative cytotoxicity of astrocytes was developed by treating them for 24 h with 0.03% H_2_O_2_.

### Transfection of Grin2c-OE Plasmid and Grin2c-SiRNA

According to the manufacturer's instructions, we used the ExFect transfection reagent (Vazyme, Nanjing, China, T101-01) to transfect Grin2c-siRNA and Grin2c-OE plasmids. Primary astrocytes (0.3–1 × 10^5^ /well) were plated in 12-well plates 24 h before transfection. For transfection preparation, 100 μL of Opti-MEM (Gibco, Grand Island, NY, USA, 31985070) was added to 1–5 μL ExFect Transfection Reagent and 1 μg plasmid or siRNA, mixed, and incubated at room temperature for 15–20 min. The transfection reagent/DNA or RNA mixture was added dropwise to astrocytes, and gently rotated to disperse evenly. At 24–48 h after transfection, cells were collected and prepared for subsequent experiments. The Grin2c-OE and Grin2c-siRNA sequences are listed in Table S1.

### Complete Spinal Cord Transection Injury Model of C57BL/6 Mice

The injury model used was that of a section of the spinal cord. To produce a mouse model of the 10^th^ thoracic vertebra (T10) complete transection SCI, C57BL/6 mice were anesthetized using 5% isoflurane in oxygen for induction and 2% isoflurane in oxygen for maintenance of anesthesia. The back fur was shaved and disinfected with Iodine Volts Swabs. An ~2 cm incision was made in the skin over T9–T11, and then the fat, fascia layer, and paravertebral muscles were separated successively to expose the T10 vertebra. T10 laminectomy was performed and T10 spinal tissue was completely transected using a sharp scalpel. In the Sham group, mice underwent the same surgical procedure except for the transection. Then, the muscle and skin were sutured layer by layer. Postoperative care was composed of disinfected wound treatment and manual bladder compression for voiding twice daily after SCI. Upon euthanasia was performed in a chamber filled with 99.9% CO_2_ gas for 10 min, and damaged regions of spinal cord tissue were harvested immediately for follow-up experiments. In particular, mice were intraperitoneally injected with an anti-mouse IL1α neutralizing antibody (R&D systems, Minneapolis, MN, SA, AF-400-NA, 20 mg/kg) and IgG control (R&D systems, Minneapolis, MN, SA, AB-108-C, 20 mg/kg) once per day for one week from the 1^st^ day after surgery.

### Extraction of mRNA and Quantitative Real-time Experiments

Quantitative real-time PCR (qRT**-**PCR) was used to determine target gene expression. First, total RNA was extracted from astrocytes and tissue using TRIzol (Invitrogen, Carlsbad, CA, USA, YZ-15596026) according to the manufacturer's instructions. Assessment of RNA yield was carried out with a Nanodrop (Thermo Fisher Scientific, Waltham, MA, USA). In the next step, total RNA was reverse transcribed to generate cDNA with the Evo M-MLV RT Premix for qPCR kit (Accurate Biology, Changsha, China, AG11706). A qRT**-**PCR was conducted with a SYBR® Green Premix Pro Taq HS qPCR Kit (Accurate Biology, Changsha, China, AG11701) according to the reagent specification. Normalized gene expression levels were calculated based on actin expression levels. The comparative CT method was used to calculate the fold change: fold change = 2^−∆∆CT^. The primer sequences for genes are listed in Table S1.

### Protein Extraction and Western Blot Experiments

Specific protein expression levels in the spinal cord of SCI mice and cells *in vitro* were assessed using Western blots (WB). A 5-mm section of the injured spinal cord centered at the epicenter of the injury site was harvested and pulverized *via* sonication and mechanical grinding to extract protein. For 30 min on ice, the cultured cells and triturated tissue were lysed with buffer [RIPA (Epizyme, Shanghai, China, PC101) + 10% PMSF (Beyotime, Shanghai, China, ST507)]. Following the collection of lysates and centrifugation, the protein concentration of the supernatants was measured, and then the samples were denatured for 5 min at 95 °C. The protein was separated on 10% acrylamide gels (Vazyme, Nanjing, China, E303-01) and transferred to polyvinylidene fluoride (PVDF) membranes (Merck, Darmstadt, Germany, ISFQ00010). The PVDF membranes were blocked with 5% skim milk for 2 h at room temperature and incubated for 12 h at 4 °C along with the following antibodies: rabbit anti-IL-1 alpha (1:1000, Proteintech, Wuhan, China, 1765-1-AP), rabbit anti-Grin2c (1:1000, Boster, Wuhan, China, PB0415), rabbit anti-CaMK2b (1:1000, Affinit, Changzhou, China, DF2907), rabbit anti-IL1-R (1:1000, Abcam, Cambridge, UK, ab229051), rabbit anti-GAPDH (1:1000, Proteintech, Wuhan, China, 10494-1-AP), and rabbit anti-beta-actin (1:1000, Proteintech, Wuhan, China, 20536-1-AP). The membranes were incubated with secondary antibodies (1:1000, Beyotime, Shanghai, China, A0208) for 1 h at room temperature. Depending on the development conditions, the appropriate developer concentration was selected and applied to the membrane, and the bands were visualized.

### Immunofluorescence Staining

The damaged region of the spinal cord was used to make frozen sections into 10 μm cryosections. Cells were grown on glass coverslips and treated as indicated. After fixation in 4% PFA, the spinal cord sections and cells were permeabilized using 0.5% Triton X-100 and blocked in 5% bovine serum albumin for 1.5 h. To obtain better immunofluorescence staining results, the samples were incubated overnight at 4°C with the following antibodies: rabbit anti-GFAP (1:200, CST, DANVERS, MA, USA, E4L7M), mouse anti-GFAP (1:300, CST, Danvers, MA, USA, GA5), rabbit anti-Grin2c (1:1000, Novus, Littleton, CO, USA, NB300-107), rabbit anti-IL1-alpha (1:250, Proteintech, Wuhan, China, 1765-1-AP), rabbit anti-Ki-67 (1:1000, Abcam, Cambridge, UK, ab15580), mouse anti-TMEM119 (1:300, CST, Danvers, MA, USA, E4B9S), rabbit anti-Nestin (1:300, CST, Danvers, MA, USA, E4O9E), rabbit anti-NeuN (1:300, CST, Danvers, MA, USA, D4G4O), and mouse anti-CSPG (1:1000, Sigma, St. Louis, MO, USA, SAB4200696). The samples were subsequently incubated with secondary antibodies (1:500, Alexa Fluor 488/647, Abcam, Cambridge, UK, ab150113/ab150079) and counterstained with DAPI (1:5000, Sigma, St. Louis, MO, USA, D9542). All immunofluorescence images were captured using a Zeiss LSM 880 confocal microscope.

We performed experiments with three or four independent mouse samples in each group. For each sample, sections from 3 levels were analyzed after identifying the damaged zone, with 3 areas per section for image observation, and the average of 9 values was recorded for statistical analysis. Regions for quantification were selected from the whole areas surrounding the wound in 3 mice per group; the regions were not divided into white or gray matter or whole ipsilateral sites.

### Measurement of Intracellular Calcium Levels

Fluo-4-acetoxymethyl ester (Fluo4) (Meilunbio, Dalian, China MA0196) was used to evaluate spontaneous intracellular Ca^2+^ transients. The membrane-permeable Fluo4 was retained in the cells and produced strong fluorescence after combining with Ca^2+^. An appropriate amount of Fluo4 solution was diluted in Hank's balanced salt solution (HBSS) (Gibco, Grand Island, NY, USA, 14175095). Then the astrocytes were incubated in the mixture away from light at 37 °C for 40 min. After washing, the cells were treated for 20 min with HBSS. Flow cytometry analysis and fluorescence microscope observation were used to detect cells and evaluate the level of intracellular Ca^2+^. Regarding the experimental design, the Blank group represents astrocytes without Fluo4 addition and the NC (negative control) group represents astrocytes treated with Fluo4 without any other treatment. The PC (positive control) group represents astrocytes with open Ca^2+^ channels as a result of the addition of 1.0 μmol/L ionomycin, an effective, selective Ca^2+^ ionophore.

### Determination of Oxidative Stress Levels

A dichlorodihydrofluorescein diacetate probe (DCFH-DA) (Beyotime, Shanghai, China, S0033S) was used to determine intracellular oxidative stress levels. As per the manufacturer's instructions, astrocytes were incubated in diluted DCFH-DA (1:1000 with serum-free DMEM) at room temperature for 20 min, shielded from light. After washing three times, the cells were detected by flow cytometry. A flow cytometry experiment was carried out to determine the ROS levels in cells by measuring the fluorescent DCF that is produced when intracellular activated oxygen oxidizes fluorescence-free DCFH-DA into fluorescent DCF.

### CCK8 Assay

The Cell Counting Kit 8 (CCK8) (Vazyme, Nanjing, China, A311-02) assay was to evaluate cell viability in the tissue culture medium through formazan, a yellow pigment produced by mitochondrial dehydrogenases. We added 10 µL of CCK-8 reagent to 96-well plates over 24 h and incubated all cells for 1 h. The absorbances of the different groups were measured with an automatic microplate reader at 450 nm.

### LDH Assay

The cytotoxicity in damaged cells was assessed by using a lactate dehydrogenase (LDH) assay kit (Bioss, Woburn, MA, USA, AK140). According to the standardized method, we extracted crude enzyme solutions from cells and tissues and sequentially added the reagents. We tested the optical density at 450 OD and calculated the LDH concentration of the samples using the standard curve. The related formulae of LDH activity are as follows: (1) Tissues: LDH (U/g) = 66.67 × LDH concentration (mol/mL)/weight(g); (2) Cells: LDH (U/10^4^ cell) = 0.133 × LDH concentration (mol/mL).

### Quantification and Statistical Analysis

Our calculations were performed with SPSS (Version 20.0, Abbott Laboratories, USA). Data are presented as the standard error of the mean (SEM) and the normalized mean value. Two-tailed Student’s *t*-tests were used to compare two independent variances to determine the statistical significance of differences. One-way analysis of variance (ANOVA) was used to compare three or more groups. Dunnett's hypothesis was applied to test for correction. A *P* value of <0.05 was considered statistically significant.

## Results

### Grin2c-Mediated Calcium Transport is Inhibited in Astrocytes after SCI According to Bioinformatic Analysis

**Fig. 1:**
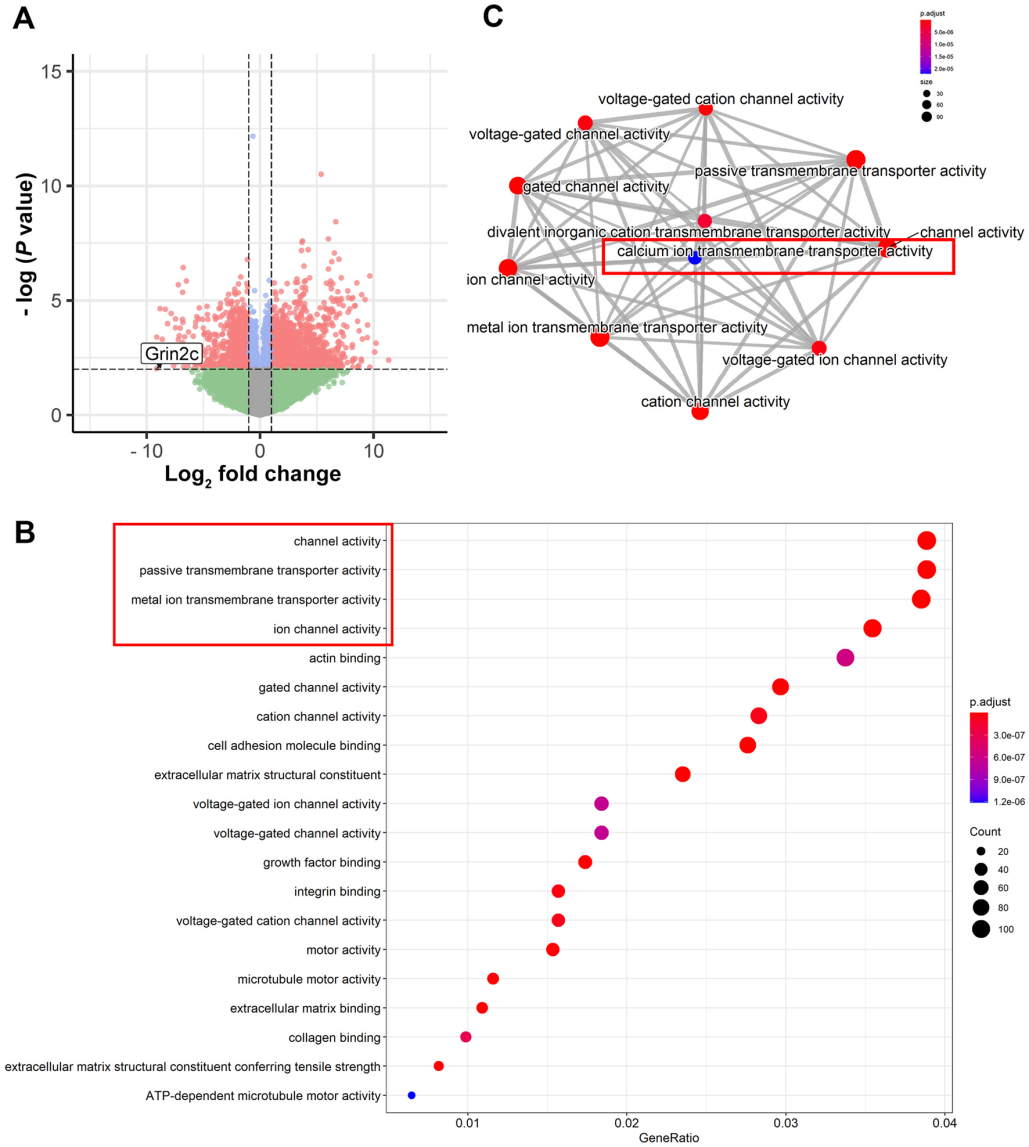
Calcium ion transmembrane transporter activity changes in astrocytes after SCI through bioinformatics analysis.** A** Transcriptome sequencing results of astrocytes in the SCI day 7 group compared with those of astrocytes in the sham group (inclusion criteria: abs (log_2_FC >1, *P*-value <0.05). There are 3173 differentially-expressed genes of astrocytes, among which 1637 increase and 1536 decrease after SCI. Grin2c expression significantly decreases after SCI (log_2_FC = –9.31860, *P*-value = 0.00945). **B** Dot plot showing the GO functional enrichment analysis results of the differential expression of genes of astrocytes after SCI (inclusion criteria: *P*-value cut-off ≤0.05, q-value cut-off ≤0.05), in which transmembrane signaling receptor activity, passive transmembrane transporter activity, channel activity, and metal ion transmembrane transporter activity significantly change. **C** Diagram showing an interactive analysis of astrocytes' GO functions. Ca^2+^ ion transmembrane transport activity is the key change in astrocyte function after SCI.

To deeply excavate the key genes of astrocytes involved in SCI, the GSE153720 dataset, a high-throughput sequencing dataset of spinal astrocytes after SCI in mice from the GEO database, was used for analysis *via* R language. In the comparison of the transcriptomes of the Sham group and the 7^th^ day after SCI (standard: abs(log_2_FC) >1, *P*-value <0.05), we found that 1637 genes were upregulated and 1536 genes were downregulated (Fig. 1A) after SCI. A total of 128 receptor-related genes (Fig. S1A) were screened out from those differentially-expressed genes, and protein-protein interaction network analysis was subsequently applied through the online String website (Fig. S1B). The results showed that 14 kinds of Ca^2+^-related glutamate receptors formed specific clusters, and their levels were mainly decreased after SCI (Fig. S1C). In particular, Grin2c was most significantly downregulated after SCI (log_2_FC= –9.31860, *P*-value = 0.00945), and was involved in metal ion transmembrane transporter activity in astrocytes following SCI (Fig. S1D). These results suggest that Grin2c may be an important SCI-related gene in astrocytes.

**Fig. 2:**
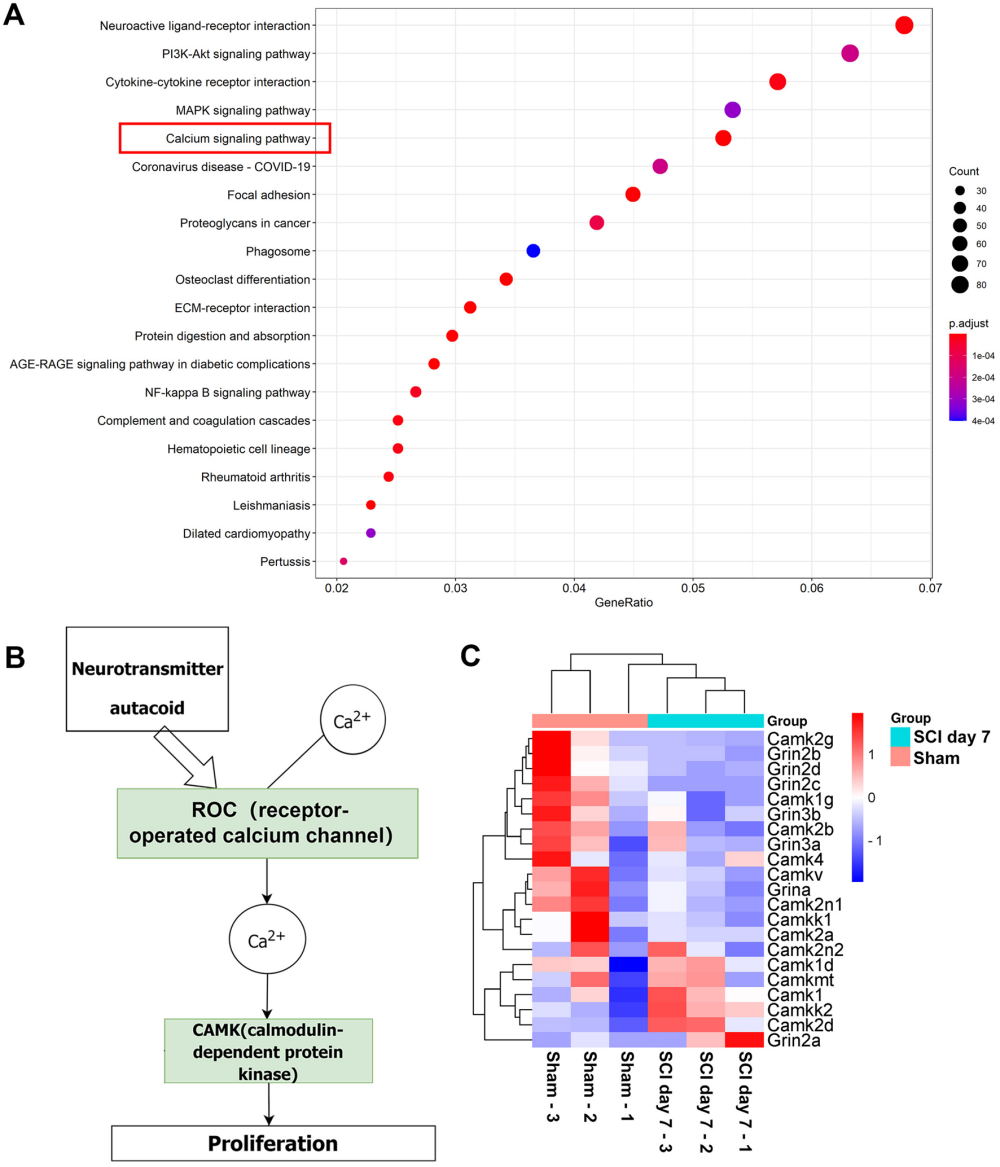
The Grin2c-mediated Ca^2+^ signaling pathway is inhibited in astrocytes after SCI through bioinformatics analysis. **A** Dot plot showing the results of KEGG pathway enrichment analysis on differentially-expressed genes of astrocytes after SCI (inclusion criteria: *P*-value cut-off ≤0.05, q-value cut-off ≤0.05), which indicates that the Ca^2+^ signaling pathways of astrocytes are significantly altered after SCI. **B** Diagram showing the various components of the astrocyte Ca^2+^ signaling pathway after SCI. Ca^2+^ channel receptors regulate intracellular Ca^2+^ and downstream CaMK to adjust cellular proliferation. **C** Heat map showing the change of components in the Ca^2+^ signaling pathway in astrocytes, and Grin2c and downstream Camk2b expression levels decrease after SCI.

To reveal the main alterations in the function of astrocytes after SCI, 3173 differentially-expressed genes were subjected to GO and KEGG analyses through clusterprofiler, GSEA, and pathview packages of R language (standard: *P*-value cutoff ≤0.05, q-value cutoff ≤0.05). According to the results of the GO functional enrichment analysis, astrocytic activity in transmembrane signaling receptors, passive transmembrane transporters, channels, and metal ion transmembrane transporters were significantly inhibited after SCI. (Fig. 1B). Importantly, the Ca^2+^ transmembrane transporter activity was the main altered astrocyte function after SCI according to the GO functional association analysis (Fig. 1C). Furthermore, KEGG analysis revealed that Ca^2+^ signaling pathways in astrocytes were significantly altered after SCI (Fig. 2A). Using Pathview analysis, the components of the Ca^2+^ signaling pathway revealed that receptor-operated Ca^2+^ channel (ROC) and CaMK specifically regulated astrocyte proliferation (Fig. 2B, Fig. S2A). The heatmap showed that the related genes of ROC and CaMK in astrocytes were downregulated after SCI (Fig. 2C). In addition, the Grin2c-related gene CaMK2b was downregulated in astrocytes after SCI (Fig. S3B, C; log_2_FC = –1.02629, *P*-value = 0.01074). Based on the bioinformatic analysis of the transcriptome of astrocytes after SCI, we showed that Ca^2+^ ion transport plays a dominant role after SCI and that the Grin2c/Ca^2+^/CaMK2b pathway may be a critical factor regulating the proliferation of astrocytes.

### The Level of Grin2c in Astrocytes Is Decreased in SCI Model Mice

**Fig. 3:**
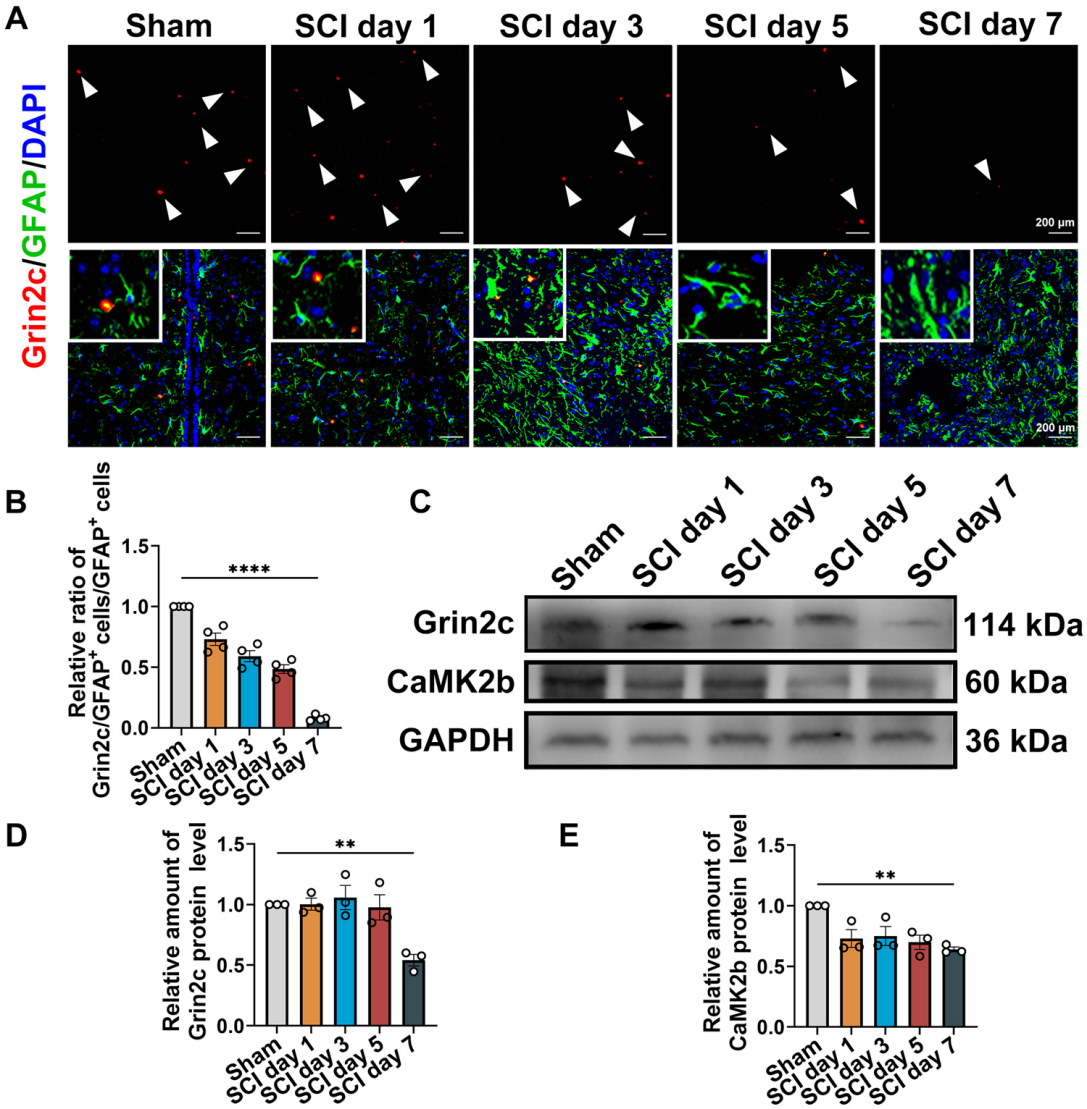
Astrocyte Grin2c levels are decreased in SCI mice. **A** Immunofluorescence staining of Grin2c/GFAP in spinal sections on different days after SCI. Scale bars, 200 μm.** B** Ratio of Grin2c^+^/GFAP^+^ cells showing that the Grin2c of astrocytes decreases after SCI. The most significant reduction of Gin2c in astrocytes was detected on the 7^th^ day following SCI. ****P <0.0001, ANOVA with Dunnett’s *post hoc* test (*n* = 4 independent animals). **C** Western blots show that the Grin2c and CaMK2b protein levels are down-regulated in the spinal cord in a time-dependent manner after injury. **D**, **E** Quantification of Grin2c and CaMK2b protein levels in the blots. Moreover, the protein levels of Grin2c (**P <0.01) and CaMK2b (**P <0.01) in the SCI day 7 group are significantly lower than those in the sham group. ANOVA with Dunnett’s *post hoc* test (*n* = 3 independent animals).

To investigate Grin2c expression in astrocytes after SCI, we constructed a T10 complete spinal cord transection model in C57BL/6 mice (Fig. S3A). After sectioning the spinal cord into continuous longitudinal sections, HE staining revealed that the spinal tissue was discontinuous and transected (Fig. S3B). Using Grin2c (red) and glial fibrillary acidic protein (GFAP) (green), we found that the density of Grin2c in astrocytes around the injury area was significantly lower in the SCI day 7 group than in the Sham group (Fig. 3A, B). In addition, WB analysis showed that the relative protein levels of Grin2c and CaMK2b were downregulated on the 7^th^ day after SCI compared to those in the Sham group (Fig. 3C–E). These results showed that the expression of Grin2c in astrocytes was downregulated in SCI model mice, consistent with the transcriptome sequencing of GSE153720.

### Grin2c Inhibits Astrocyte Proliferation by Increasing Intracellular Ca^2+^ and CaMK2b Levels ***in vitro***

**Fig. 4:**
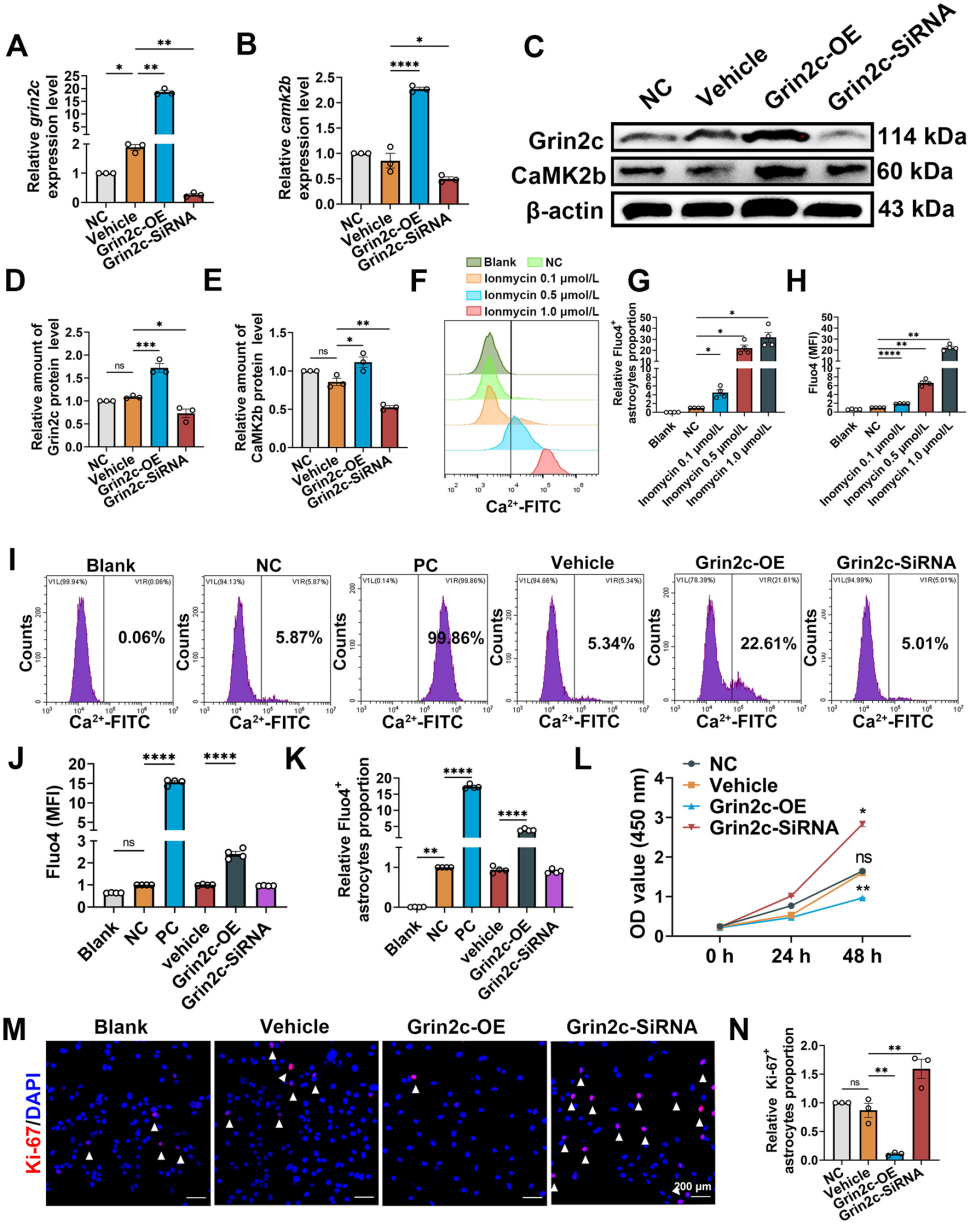
Grin2c inhibits the proliferation of astrocytes through the Ca^2+^/CaMK2b pathway *in vitro*. **A**, **B** Expression of Grin2c and CaMK2b in the transfected astrocytes as detected by qRT–PCR. Compared with the Vehicle group, the relative levels of *grin2c* and *camk2b* are increased in the Grin2c-OE group and decreased in the Grin2c-SiRNA group. ****P <0.0001, **P <0.01, *P <0.05, ANOVA with Dunnett’s *post hoc* test (*n* = 3 biological replicates).** C–E** Western blots and analysis showing the protein levels of Grin2c and CaMK2b in the Grin2c-OE group and Grin2c-SiRNA group, and the trend of protein changes is consistent with that of mRNA. ***P <0.001, **P <0.01, *P <0.05, ANOVA with Dunnett’s *post hoc* test (*n* = 3 biological replicates).** F**–**H** Flow cytometry and quantification of intracellular Ca^2+^ influx, detected using the fluorescent Ca^2+^ indicator Fluo4. After opening the membrane Ca^2+^ channel with ionomycin, the mean fluorescence intensity (MFI) and Fluo4^+^ cells' relative proportion of astrocytes are significantly increased. **I**–**K** After overexpression of Grin2c, the intracellular Ca^2+^ level of astrocytes is upregulated. ****P <0.0001, ANOVA with Dunnett’s *post hoc* test (*n* = 4 biological replicates). **L** Cell viability was assessed by CCK8 assays, and the inhibition of Grin2c is also shown to increase the activity of astrocytes (48 h Grin2c-OE *vs* 48 h Vehicle, **P <0.01; 48 h Grin2c-SiRNA *vs* 48 h Vehicle *P <0.05), ANOVA with Dunnett’s *post hoc* test (*n* = 4 biological replicates).** M** Immunohistochemistry of astrocytes using a Ki-67 antibody displays nuclear Ki-67 immunoreactivity, a marker of cell proliferation. Scale bars, 200 μm, ANOVA with Dunnett’s *post hoc* test (*n* = 3 biological replicates).** N** The relative proportion of Ki-67^+^ astrocytes in all cells. The proportion of proliferating astrocytes decreases after overexpression, while the proportion of astrocytes proliferating increases after interfering with Grin2c. **P <0.01, ANOVA with Dunnett’s *post hoc* test (*n* = 3 biological replicates).

To clarify the specific roles of Grin2c in astrocyte proliferation, we isolated astrocytes from neonatal mouse brains (Fig. S3C, D) and constructed separate transfection models using a Grin2c overexpression plasmid (Grin2c-OE) group and a Grin2c SiRNA (Grin2c-SiRNA) group (Table S1). In the Grin2c-OE group, the relative messenger ribonucleic acid (mRNA) levels of Grin2c and CaMK2b were well overexpressed (Fig. 4A, B), which indicates that the transfection model was successfully constructed and that Grin2c can affect CaMK2b expression. Compared to astrocytes treated with only vehicle (Vehicle group), the Grin2c-OE group exhibited an increase relative Grin2c and CaMK2b protein levels, whereas Grin2c-SiRNA transfection decreased them (Fig. 4C–E). Fluo4 was used to monitor changes in intracellular Ca^2+^ ion concentrations in astrocytes. After stimulation with ionomycin, an effective Ca^2+^ ionophore, the mean fluorescence intensity (MFI), and the proportion of Fluo4^+^ astrocytes were significantly increased, as determined by flow cytometry (Fig. 4F–H). Ionomycin treatment effectively increased the intracellular Ca^2+^ level of astrocytes and was regarded as a positive control for intracellular Ca^2+^ estimation. As shown in Fig. 4I–K, overexpression of Grin2c increased the intracellular Ca^2+^ ion concentration in astrocytes. The CCK8 results showed that Grin2c inhibition increased the activity of astrocytes, whereas Grin2c overexpression inhibited astrocyte proliferation (48 h Grin2c-OE *vs* 48 h Vehicle, **P <0.01) (48 h Grin2c-SiRNA *vs* 48 h Vehicle *P <0.05) (Fig. 4L). Next, astrocyte proliferative ability was evaluated by staining with the mitosis-related antibody Ki-67, and the results showed that the Grin2c-SiRNA group had a significant increase in proliferative ability compared to that in the vehicle group (Fig. 4M, N).

In summary, Grin2c is a proliferation-suppressor gene in astrocytes and its overexpression can decrease the proliferation of astrocytes through the Ca^2+^/CaMK2b pathway *in vitro*.

### IL1α Can Reduce the Level of Grin2c and Calcium Signaling Pathways in Astrocytes *in vitro*

**Fig. 5:**
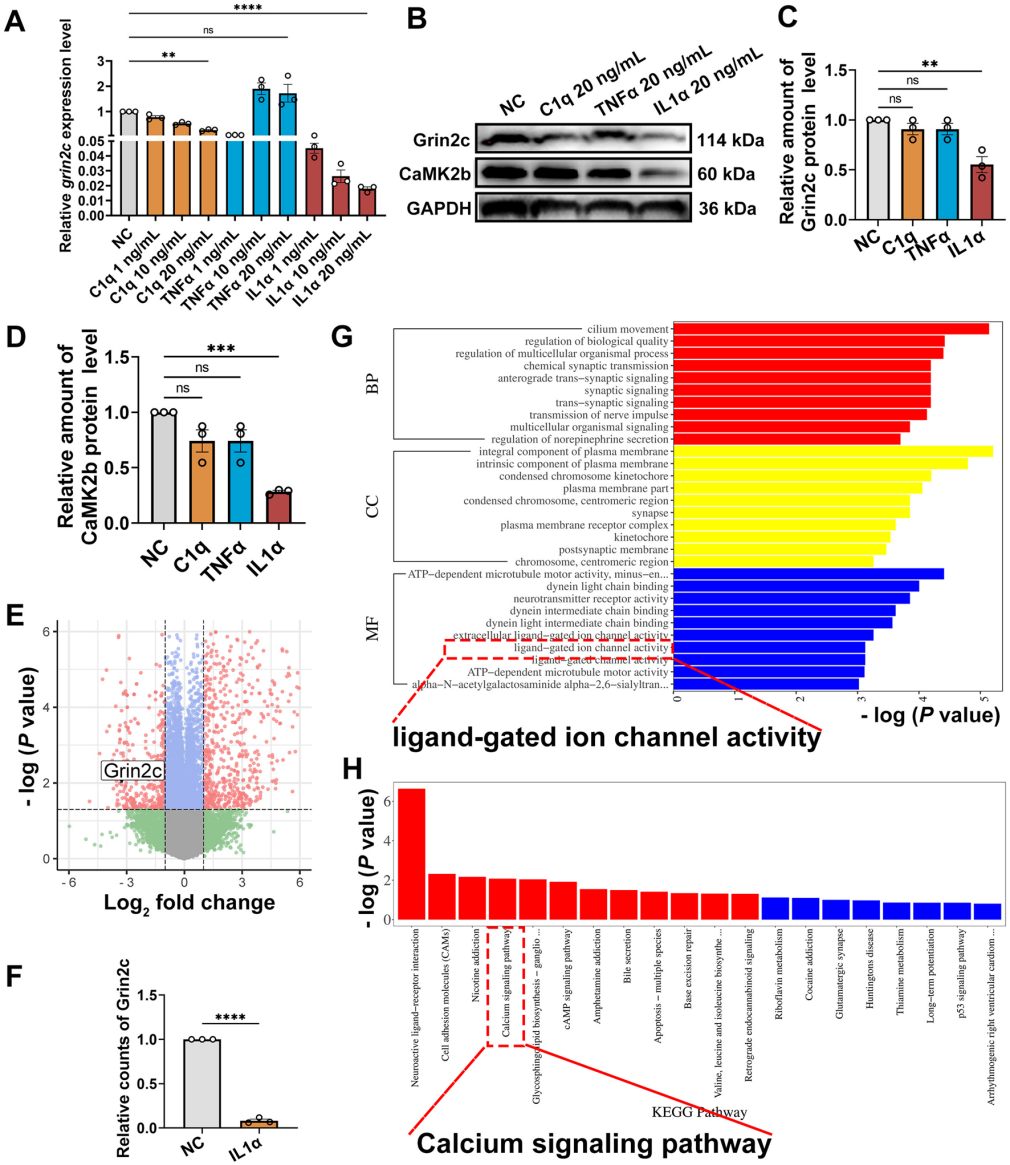
IL1α screened from other inflammatory factors downregulates Grin2c and Ca^2+^ signaling in astrocytes. **A** Quantitative real-time polymerase chain reaction (qRT-PCR) analysis reveals the relative *grin2c* mRNA level in astrocytes after 24 h stimulation with IL1α, C1q, and TNFα. Compared with the 20 ng/mL C1q group, the 20 ng/mL IL1α group shows more evident downregulation compared to the negative control group. And 20 ng/mL TNFα up-regulates Grin2c mRNA levels in astrocytes. ****P <0.0001, **P <0.01, ANOVA with Dunnett’s *post hoc* test (*n* = 3 biological replicates). **B** Western blots of Grin2c and CaMK2b after IL1α 20 ng/mL, C1q 20 ng/ml, and TNFα 20 ng/mL stimulation for 24 h; the trend of protein changes is consistent with that of mRNA. **C**, **D** Quantification of Grin2c and CaMK2b. ***P <0.001, **P <0.01, ANOVA with Dunnett’s *post hoc* test (*n* = 3 biological replicates). **E** Transcriptome sequencing of astrocytes in the IL1α group (20 ng/mL IL1α, 24 h) compared with those of astrocytes in the NC group (inclusion criteria: abs (log_2_FC >1, *P*-value <0.05). There are 1998 differentially-expressed genes of astrocytes, among which 1001 are increased and 997 are decreased after IL1α stimulation. **F** Grin2c is significantly reduced in the IL1α group in counts data from high-throughput sequencing. ****P <0.0001, ANOVA with Dunnett’s *post hoc* test (*n* = 3 biological replicates).** G** Bar graph showing the top 10 GO functional enrichment analysis results of biological process (BP), cellular component (CC), and molecular function (MF) (inclusion criteria: *P*-value cut-off ≤0.05, q-value cut-off ≤0.05), in which ligand-gated ion channel activity and neurotransmitter receptor activity significantly change. **H** Bar graph showing the KEGG pathway enrichment analysis results of the differentially-expressed genes of astrocytes after IL1α stimulation (inclusion criteria: *P*-value cut-off ≤0.05, q-value cut-off ≤0.05), in which the Ca^2+^ signaling pathway significantly changes.

During the early stages of SCI, inflammatory factors in the damaged environment activate and proliferate astrocytes abnormally, and the key inflammatory factors include IL1α, C1q, and TNFα [5]. To investigate the specific inflammatory factor responsible for enhancing astrocyte proliferation by inhibiting Grin2c, astrocytes were stimulated for 24 h *in vitro* with different concentrations of recombinant IL1α, C1q, and TNFα proteins. Compared with the 20 ng/mL C1q group, the 20 ng/mL IL1α group showed more evident downregulation of Grin2c mRNA compared to that in the negative control group (Fig. 5A). Compared to the other two factors, IL1α significantly decreased the relative Grin2c protein levels and downstream CaMK2b levels (Fig. 5B–D). WB results showed that the corresponding receptor of IL1α was expressed in astrocytes, which was consistent with microglia (Fig. S4A) [30].

To further assess the alteration of astrocytes after IL1α stimulation, high-throughput transcriptome sequencing was conducted to compare the differences between NC astrocytes and the IL1α-treated astrocytes (20 ng/mL IL1α stimulation for 24 h) (standard: abs(log_2_FC) >1, *P*-value <0.05). A total of 1998 differentially-expressed genes were identified, 1001 of which were upregulated and 997 of which were downregulated, and Grin2c was significantly downregulated in response to IL1α stimulation (Fig. 5E, F). According to the GO enrichment analysis, astrocytes could be reactive *via* interleukins (Fig. S4B). Ligand-gated ion channel function in astrocytes was markedly altered after IL1α stimulation (Fig. 5G). Further KEGG analysis revealed alterations in the Ca^2+^ signaling pathway (Fig. 5H), which may be involved in neuroactive ligand-receptor interactions (Fig. S4C). The results of GO and KEGG analyses presented the potential function of IL1α in the Grin2c-mediated Ca^2+^ pathway. In summary, IL1α can downregulate Grin2c and the Ca^2+^ signaling pathway in astrocytes *in vitro*.

### IL1α Induces Astrocyte Proliferation by Suppressing the Grin2c/Ca^2+^/CaMK2b Pathway in an ***in Vitro*** Oxidative Damage Model

We investigated the role of the IL1α/Grin2c/Ca^2+^/CaMK2b pathway in astrocyte proliferation under SCI injury conditions, such as ischemia and hypoxia, ROS injury, and an inflammatory cascade reaction. We simulated the SCI microenvironment to construct *in vitro* injury models: the OGD model, the oxidative damage model, and the inflammatory model (Fig. S5A). Astrocyte viability was reduced in the OGD model, the oxidative injury model, and the inflammation model (Fig. S5B). The cytotoxicity in the OGD model and the oxidative damage treatment conditions was significantly higher than that of the control condition (Fig. S5C). Compared to that in the NC group, the level of ROS in the OGD group and the oxidative damage group significantly increased (Fig. S5D–E). In summary, simulating the unfavorable conditions of SCI using the OGD model and the oxidative damage model was equally successful. The intracellular Ca^2+^ level of astrocytes increased in the OGD model group and oxidative damage model group (Fig. S5F–J). However, compared with the OGD model, the oxidative damage model increased the levels of Grin2c and CaMK2b (Fig. S5K–M). It appears that the inhibitory effect of the oxidative damage model on the proliferation of astrocytes may be related to the upregulation of Grin2c expression. Thus, for the next investigation, we chose the oxidative damage model, since it is more conducive to determining whether IL1α induces the proliferation of astrocytes under adverse environmental conditions by inhibiting Grin2c expression.

**Fig. 6:**
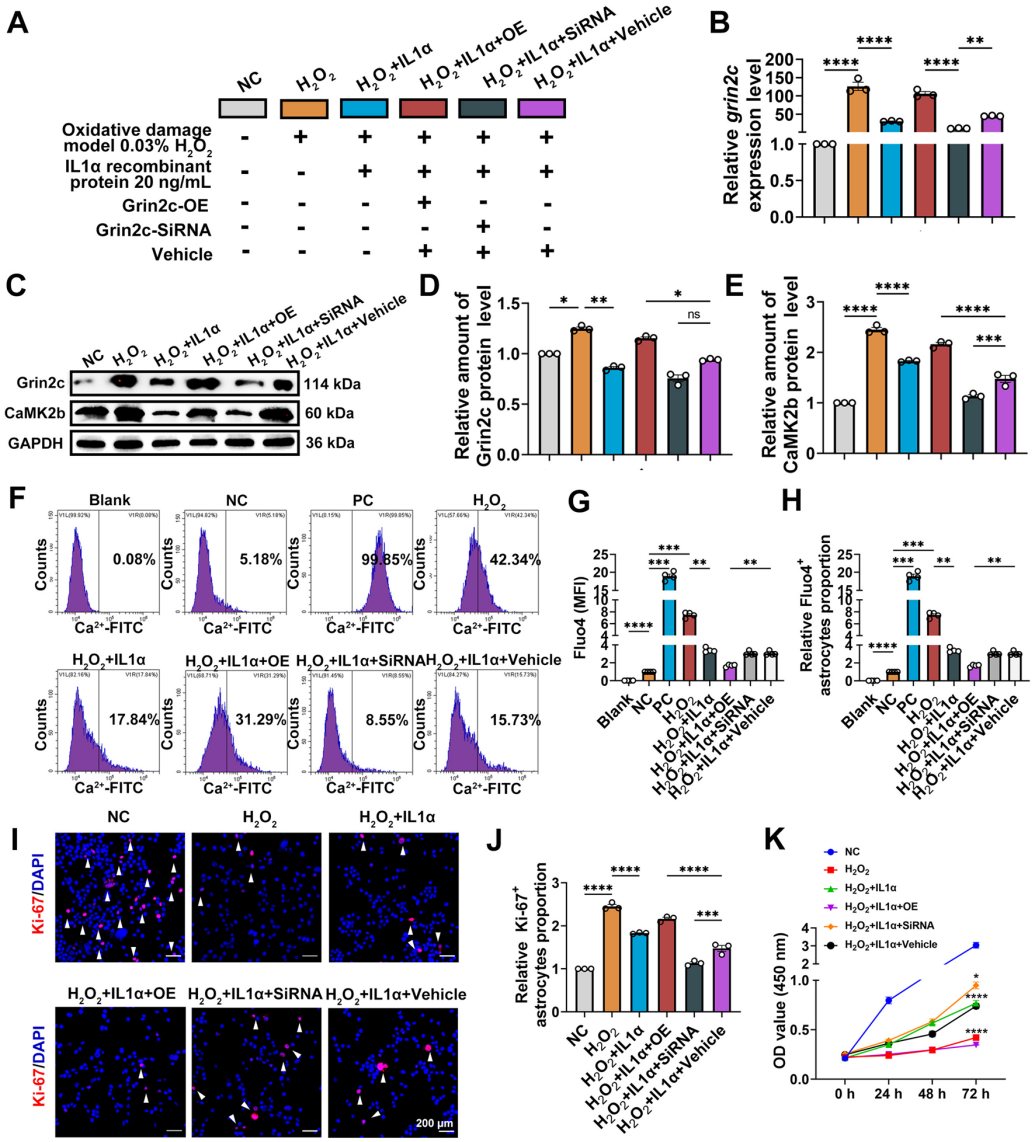
IL1α induces astrocyte proliferation through suppression of the Grin2c/Ca^2+^/CaMK2b pathway in the *in vitro* oxidative damage model. A Groups set as an annotation set. **B** The relative grin2c mRNA expression level was assessed by qRT-PCR. The Grin2c level is decreased after IL1α stimulation. Overexpression of Grin2c restores the inhibitory effect of IL1α on Grin2c in astrocytes. ****P <0.0001, **P <0.01, ANOVA with Dunnett’s *post hoc* test (*n* = 3 biological replicates).** C** Protein levels of Grin2c and CaMK2b detected by WB assay. **D**, **E** Quantification of Grin2c and CaMK2b protein levels. Compared with the NC group, the Grin2c and CaMK2b levels in the H_2_O_2_ group were significantly increased, but decreased after the addition of IL1α. Meanwhile, overexpression of Grin2c restores the inhibitory effect of IL1α on CaMK2b in astrocytes. ****P <0.0001, **P <0.01, ANOVA with Dunnett’s *post hoc* test (*n* = 4 biological replicates). **F**–**H** Flow cytometry and quantification of intracellular Ca^2+^ influx. Compared with the NC group, the intracellular Ca^2+^ level in the H_2_O_2_ group is significantly increased but decreases after the addition of IL1α. Meanwhile, overexpression of Grin2c restores the inhibitory effect of IL1α on intracellular Ca^2+^ levels in astrocytes. ****P <0.0001, ***P <0.001, **P <0.01, ANOVA with Dunnett’s *post hoc* test (*n* = 3 biological replicates). **I** Immunohistochemistry of Ki-67 staining. Scale bars, 200 μm.** J** Quantification of Ki-67^+^ astrocyte ratio. Compared with the NC group, the proliferation of the H_2_O_2_ group is significantly decreased, and the proliferation is restored after IL1α stimulation. Overexpression of Grin2c also inhibits the proliferation of astrocytes. ****P <0.0001, ***P <0.001, ANOVA with Dunnett’s *post hoc* test (*n* = 3 biological replicates). **K** Cell viability was assessed by CCK8 assays, and the changing trend is consistent with Ki-67 staining. ****P <0.0001, *P <0.05, ANOVA with Dunnett’s *post hoc* test (*n* = 4 biological replicates).

To further investigate the role of IL1α in Grin2c and astrocyte proliferation in the oxidative damage model, the experimental groups were set up as follows: NC (negative control), H_2_O_2_ (oxidative damage model), H_2_O_2_+IL1α, H_2_O_2_+IL1α+Grin2c-OE, H_2_O_2_+IL1α+Grin2c-SiRNA, and H_2_O_2_+IL1α+vehicle (Fig. 6A). Compared with that in the NC group, the relative Grin2c mRNA level significantly increased in the H_2_O_2_ group, and IL1α inhibited the increase in Grin2c levels in the oxidative damage model. The relative Grin2c mRNA level was also markedly increased by H_2_O_2_+IL1α+Grin2c-OE treatment or decreased in the H_2_O_2_+IL1α+Grin2c-SiRNA group in comparison to the H_2_O_2_+IL1α+vehicle group (Fig. 6B). Based on the WB results, we found that IL1α significantly suppressed the elevation of Grin2c and CaMK2b protein levels in the oxidative damage model. Grin2c overexpression reversed the inhibitory effect of IL1α on Grin2c and promoted CaMK2b upregulation (Fig. 6C–E). Moreover, IL1α inhibited the level of Ca^2+^ influx by oxidative damage in astrocytes, and the intracellular Ca^2+^ levels of the H_2_O_2_+IL1α+Grin2c-OE group were increased compared with those of the H_2_O_2_+IL1α+vehicle group (Fig. 6F–H). As part of the study, immunofluorescence staining was used to determine the Ki-67^+^ astrocyte ratio in oxidative injury models and assess astrocyte proliferation (Fig. 6I–J). In comparison with that of the NC group, the proliferation of the H_2_O_2_ group was significantly decreased, but the proliferation was restored after the addition of IL1α. Furthermore, overexpression of Grin2c inhibited the proliferation of astrocytes stimulated by IL1α. CCK8 assays were applied to test the proliferation of astrocytes (Fig. 6K). The results showed the change was consistent with Ki-67 staining (72 h NC *vs* 72 h H_2_O_2_, ****P <0.0001; 72 h H_2_O_2_ *vs* 72 h H_2_O_2_+IL1α, ****P <0.0001; 72 h H_2_O_2_+IL1α+Grin2c-OE *vs* 72 h H_2_O_2_+IL1α+Vehicle, ****P <0.0001; 72 h H_2_O_2_+IL1α+Grin2c-SiRNA *vs* 72 h H_2_O_2_+IL1α+Vehicle, *P <0.05). In the presence of IL1α and H_2_O_2_, overexpression of Grin2c decreased the proliferative capacity of astrocytes as compared to that of the vehicle group. In summary, IL1α can regulate the proliferation of astrocytes *via* the Grin2c/Ca^2+^/CaMK2b pathway in a model of oxidative damage.

### Blockade of IL1α Using Neutralizing Antibodies Enhances Grin2c Levels and Suppresses Astrocyte Proliferation in SCI Model Mice

To define the role of IL1α in astrocytes *in vivo*, we evaluated the IL1α level in the lesions of SCI model mice. Immunofluorescence showed that IL1α expression was significantly upregulated in the lesion at 5 days after SCI (Fig. S6A, B), as well as the protein level of IL1α in the injured spinal tissue (Fig. S6C, D). To monitor the proliferation of astrocytes after SCI, a Ki-67/GFAP^+^ immunofluorescence assay was conducted to determine the proportion of astrocytes proliferation around the damaged region (Fig. S6E, F). The results showed that astrocytes proliferated after SCI, and the levels of IL1α were increased in the injury area.

**Fig. 7:**
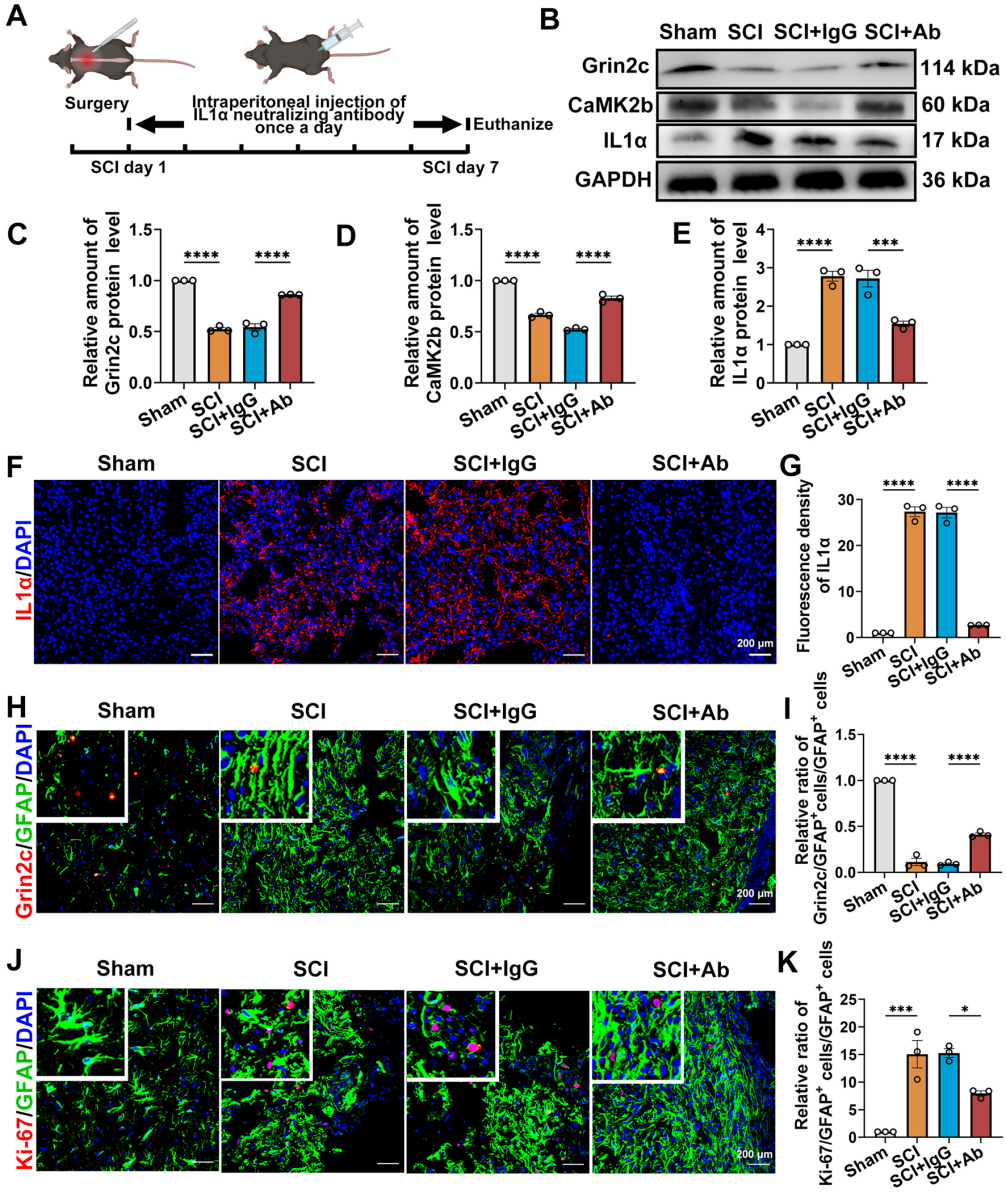
Blockade of IL1α using neutralizing antibodies enhances the Grin2c of astrocytes and suppresses astrocyte proliferation in SCI mice.** A** Schematic of intraperitoneal neutralizing antibody injection after SCI. **B** Protein levels of Grin2c, IL1α, and CaMK2b in the injured spinal cord as determined by WB assays after injection of neutralized antibody. **C**–**E** Quantitative analysis of IL1α, Grin2c, and CaMK2b protein levels in WB assays. The level of IL1α in the spinal cord is significantly decreased by injection of IL1α neutralizing antibody compared with the SCI+IgG group (injection control group). The levels of Grin2c and CaMK2b in the spinal cord are significantly increased after the blockade of IL1α. ****P <0.0001, ***P <0.001, ANOVA with Dunnett’s post hoc test (*n* = 3 independent animals). **F**, **G** Immunofluorescence staining and quantitative analysis of IL1α in the injured area after injection of IL1α neutralizing antibody after SCI. The results show a significantly decreased level of IL1α in the injured environment after injection of neutralizing antibody. Scale bars, 200 μm, ****P <0.0001, ANOVA with Dunnett’s *post hoc* test (*n* = 3 independent animals).** H** Grin2c/GFAP immunofluorescence staining of spinal sections after injection of neutralizing antibody. Scale bars, 200 μm. **I** Grin2c^+^/GFAP^+^ cell ratios showing that Grin2c of astrocytes is upregulated after blockade of IL1α in SCI mice, ****P <0.0001, ANOVA with Dunnett’s *post hoc* test (*n* = 3 independent animals). **J** Immunofluorescence staining of Ki-67/GFAP around damage following the blockade of IL1α. Scale bars, 200 μm. **K** Ki-67^+^/GFAP^+^ cell ratios show that neutralizing IL1α effectively inhibits the proliferation of astrocytes after SCI. ***P <0.001, *P <0.05, ANOVA with Dunnett’s *post hoc* test (*n* = 3 independent animals).

To further directly identify the role of IL1α in astrocytes after SCI, mice were administered 20 mg/kg of anti-IL1α-neutralizing antibodies once per day for one week after surgery (Figs 7A and S7A). Compared with the SCI+IgG group, the level of IL1α around the lesion was decreased in the IL1α neutralizing antibody group after SCI (Fig. 7B, E, F, and G). It has been shown that neutralizing antibodies reduce the levels of IL1α in areas of injury. Grin2c and CaMK2b protein levels were also increased in the SCI+Ab group (Fig. 7B–D). As shown by immunofluorescence labeling of Grin2c^+^/GFAP^+^ cells, astrocyte Grin2c levels were increased following suppression of IL1α after SCI (Fig. 7H, I). Due to the low level of IL1α in the sham group, IL1α neutralizing antibodies had little effect on the ILα/Grin2c/CaMK2b pathway in the astrocytes of the Sham+Ab group (Fig. S6B–I). In addition, immunofluorescence staining and quantitative analysis of the Ki-67^+^/GFAP^+^ cells showed a low proportion of these cells after IL1α neutralization, which indicated that IL1α administration increases astrocyte proliferative activity (Fig. 7J, K). Compared with that in the Sham+IgG group, neutralizing IL1α did not effectively influence the proliferation of astrocytes in sham+Ab mice (Fig. S7J, K). Furthermore, HE staining showed that the lesion volume of spinal tissue was decreased after injection of IL1α neutralizing antibodies (Fig. S8A, B). Immunofluorescence staining of CSPG (chondroitin sulfate proteoglycans)/GFAP in the damaged area after SCI showed that the lesion scar was significantly decreased after blockade of IL1α in SCI mice (Fig. S8C–E).

This evidence supports the conclusion that blockade of IL1α in the damaged region reduces astrocyte proliferation inhibits Grin2c expression in the astrocytes of SCI model mice, and decreases the lesion scar and lesion volume in these mice.

## Discussion

**Fig. 8:**
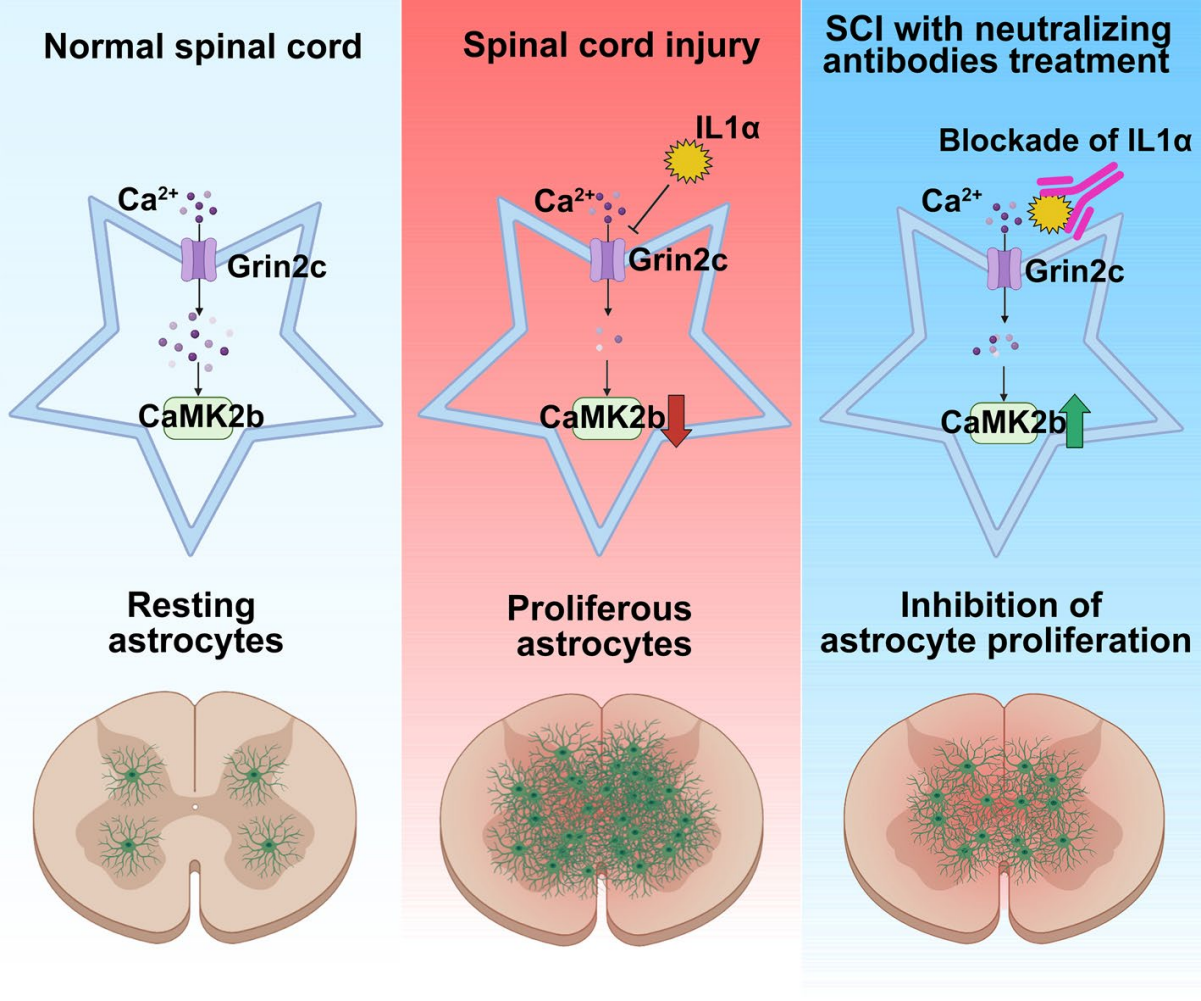
Schematic showing that after spinal cord injury, IL1α induces astrocyte proliferation by inhibiting Grin2c, resulting in decreased intracellular Ca^2+^ concentration and CaMK2b levels. In response to the blockade of IL1α *via* neutralizing antibodies, Grin2c/Ca^2+^/CaMK2b pathway levels increase, and astrocyte proliferation decreases.

SCI causes considerable physical and psychological harm to patients and imposes financial burdens on families and society [31]. Recently, many neurorestorative strategies have been tested to repair those damaged functions and/or structures [32–35], especially virtual reality technology has been used to assist the study of the neural mechanisms that underlie behaviors modulated by the environmental context [36]. After SCI, an abnormal and continuously active inflammatory response causes substantial neuronal death [37]. Moreover, astrocytes might be specifically activated *via* the inflammatory factors IL1α, C1q, and TNFα, secreted by microglia [5]. Inhibition of microglia after SCI reduces astroglial scar formation [38], and knockout of microglia-mediated inflammatory factors inhibits astrocyte neurotoxic action [39]. By screening these factors, we found that IL1α effectively inhibited Grin2c expression and induced abnormal astrocyte proliferation after SCI. In brain injury, IL1α also induces the activation of glial cells [40]. IL1α can stimulate astrocytes to produce ROS and stimulate oligodendrocyte death [41]. Thus, IL1α appears to be involved in neurotrauma-related astrocyte activity. In other models of neurotoxicity, IL1α expression levels increase and NMDAR expression levels decrease [42]. In our study, an intraperitoneal injection [43] of neutralizing antibodies for 7 days reduced the level of IL1α and decreased the proliferation of astrocytes and lesion scar/volume in SCI model mice. Furthermore, Ab treatment may also alleviate microglia-mediated inflammation and deserves to be further explored in future studies (Fig. 8).

As the receptor of the predominant excitatory neurotransmitter, Grin2c plays an essential role in abnormal cell proliferation [44–46]. Grin2c is also related to brain development, as it promotes neuronal integrity, neurogenesis, and synaptic remodeling [47–50]. Moreover, numerous organic and non-organic disorders in the CNS have been linked to Grin2c, such as alcohol withdrawal syndrome [51], schizophrenia [52], mood disorder [53], degenerative lumbar scoliosis [54], and Parkinson's disease [55]. In addition, grin2c levels have been shown to decline following traumatic brain injuries, which is consistent with our study on SCI [56]. In recent studies on Grin2c, there has been some uncertainty regarding the relationship between Grin2c and astrocytes. Based on bioinformatic analysis and experiments in our study, reduced Grin2c enhances astrocyte proliferation *via* the Ca^2+^/CaMK2b pathway after SCI. These results show that Grin2c, as a growth-regulating factor in astrocytes, contributes to the pathology of SCI. Based on the proton electrochemical potential gradient, the highly specific and sensitive Ca^2+^-permeable NMDAR promotes Ca^2+^ ion influx [57–59]. The NMDARs are tetrameric ligand-gated Ca^2+^ ionotropic receptors that are activated by glutamate [60] and mediate neural excitotoxicity *via* Ca^2+^-dependent glutamate release in diseases of the CNS [61]. The NMDARs of astrocytes also play important roles in neurotransmission and homeostasis [36]. It is imperative to note that neurons are killed by cell edema in early ischemic brain injury through hyperactivation of NMDARs [62] and that NMDAR expression levels increase due to oxidative stress [63]. Unlike neurons, the low levels of Grin2c induced by IL1α enhanced the proliferation of astrocytes after SCI in our research. This process could explain why astrocytes proliferate under adverse conditions in damaged areas after SCI.

In contrast to damage models impairing the survival of astrocytes *in vitro* [64–66], the microenvironment of CNS injury causes reactive astrocytes to proliferate under challenging circumstances [3, 67, 68]. This discrepancy is mainly a result of the major disadvantages of *in vivo* models, such as the lack of natural immune systems and, especially, the lack of systemic interaction with other cells. In our study, we identified IL1α as the key regulator of astrocyte proliferation in the oxidative damage model, and blockade of IL1α decreased the proliferation of astrocytes in the complete transection SCI model [69]. Moreover, contusion injury is the most common type of SCI in clinical settings [70]. To ensure the consistency of the degree of injury and improve the accuracy of the later histological evaluation, we chose the complete transection model. In future studies, we will enhance our work using contusion models, since they are more clinically relevant. In the early phase of SCI, astrocyte proliferation reduces inflammation and preserves neural tissue [71]. However, the abnormal proliferation of astrocytes results in the formation of astrocyte scars, which may replace normal neural tissue and inhibit the regeneration of axons in the course of the process [72]. The dysfunction of astrocyte proliferation, termed “reactive astrogliosis”, is a common response to all CNS injuries/diseases [73].

In conclusion, we found that the decreased level of Grin2c induced astrocyte proliferation by reducing intracellular Ca^2+^ levels and CaMK2b expression levels in astrocytes after SCI. And IL1α can inhibit the Grin2c/Ca^2+^/CaMK2b pathway to induce astrocyte proliferation and scar formation. Blockade of IL1α may be a novel therapeutic strategy for ameliorating SCI in the future.

### Supplementary Information

Below is the link to the electronic supplementary material.

Supplementary file1 (PDF 2552 kb)

## Data Availability

All the data supporting this study are available in the manuscript and its Supplementary Information file. Raw data are available from the corresponding authors upon reasonable request.
