# Supplementary material for: Inflammatory Factor IL1α Induces Aberrant Astrocyte Proliferation in Spinal Cord Injury Through the Grin2c/Ca2+/CaMK2b Pathway
Source: Neurosci Bull. 2023 Oct 21;40(4):421–38. doi: 10.1007/s12264-023-01128-4 (PMC11003951; doi:10.1007/s12264-023-01128-4)
Supplement: Supplementary file 1 — Supplementary file1 (PDF 2552 kb) [file 12264_2023_1128_MOESM1_ESM.pdf]

Supplementary Materials

Supplemental Figures and Figure Legends

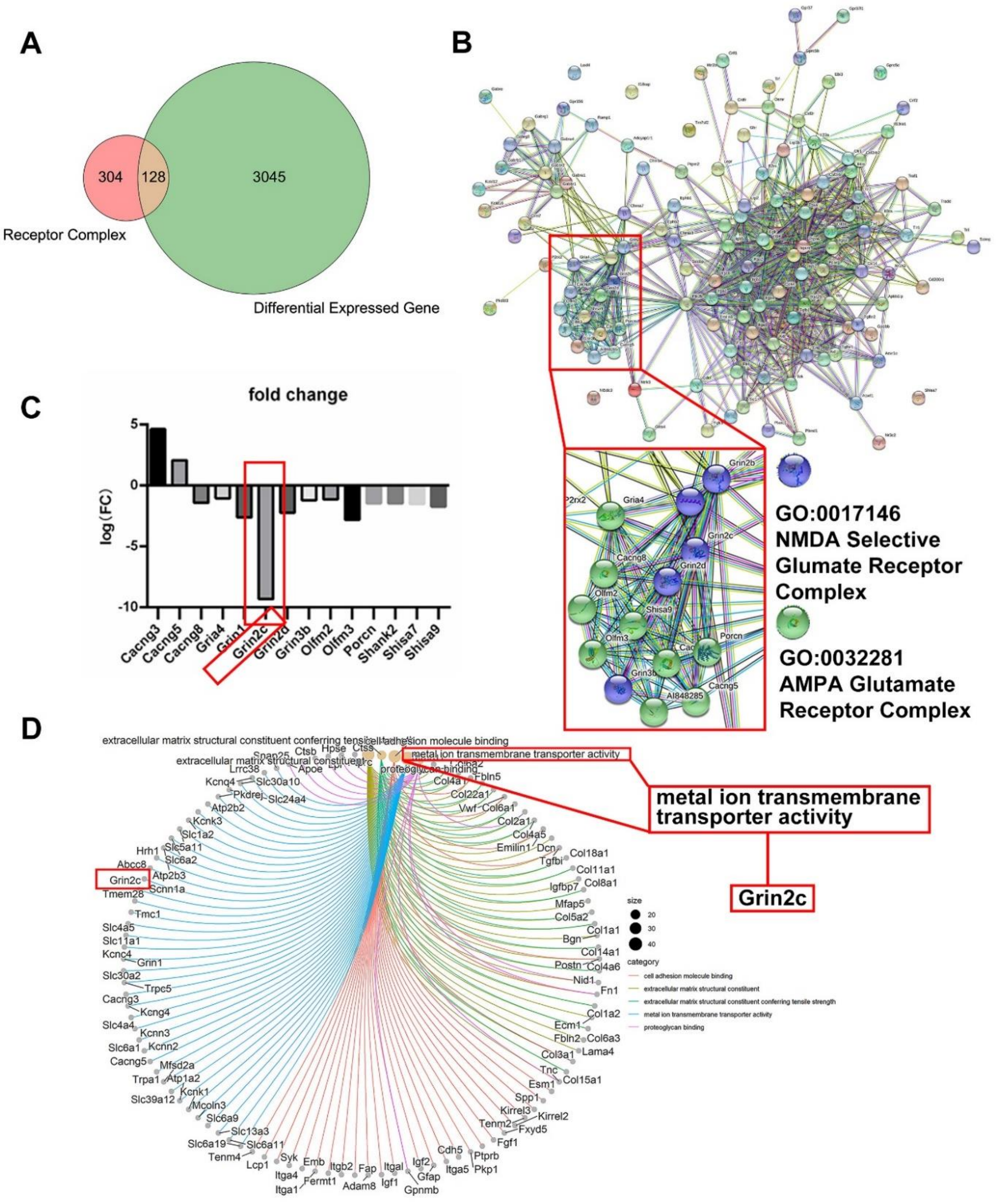

Fig. S1 Grin2c expression is reduced in astrocytes after SCI. **A** Venn diagram analysis showing the 128 intersecting genes between the differentially-expressed genes after SCI and receptor complex (GO:0043235), and 128 differentially-expressed genes related to receptors. **B** The protein interaction network of 128 intersecting genes generated using the online String website, and the String analysis results show that 14 Ca^{2+}-related glutamate receptor genes form a cluster of specific protein interactions. **C** The expression levels of these genes are significantly decreased after SCI, suggesting that the level of glutamate receptors in astrocytes decreases after SCI. And Grin2c decreases most significantly ($\log_2$FC = –9.31860, *P*-value = 0.00945). This suggests that Grin2c may be a target gene for astrocytes after SCI. **D** As shown in the diagram, Grin2c is one of the genes involved in metal ion transmembrane transporter activity in astrocytes following SCI.

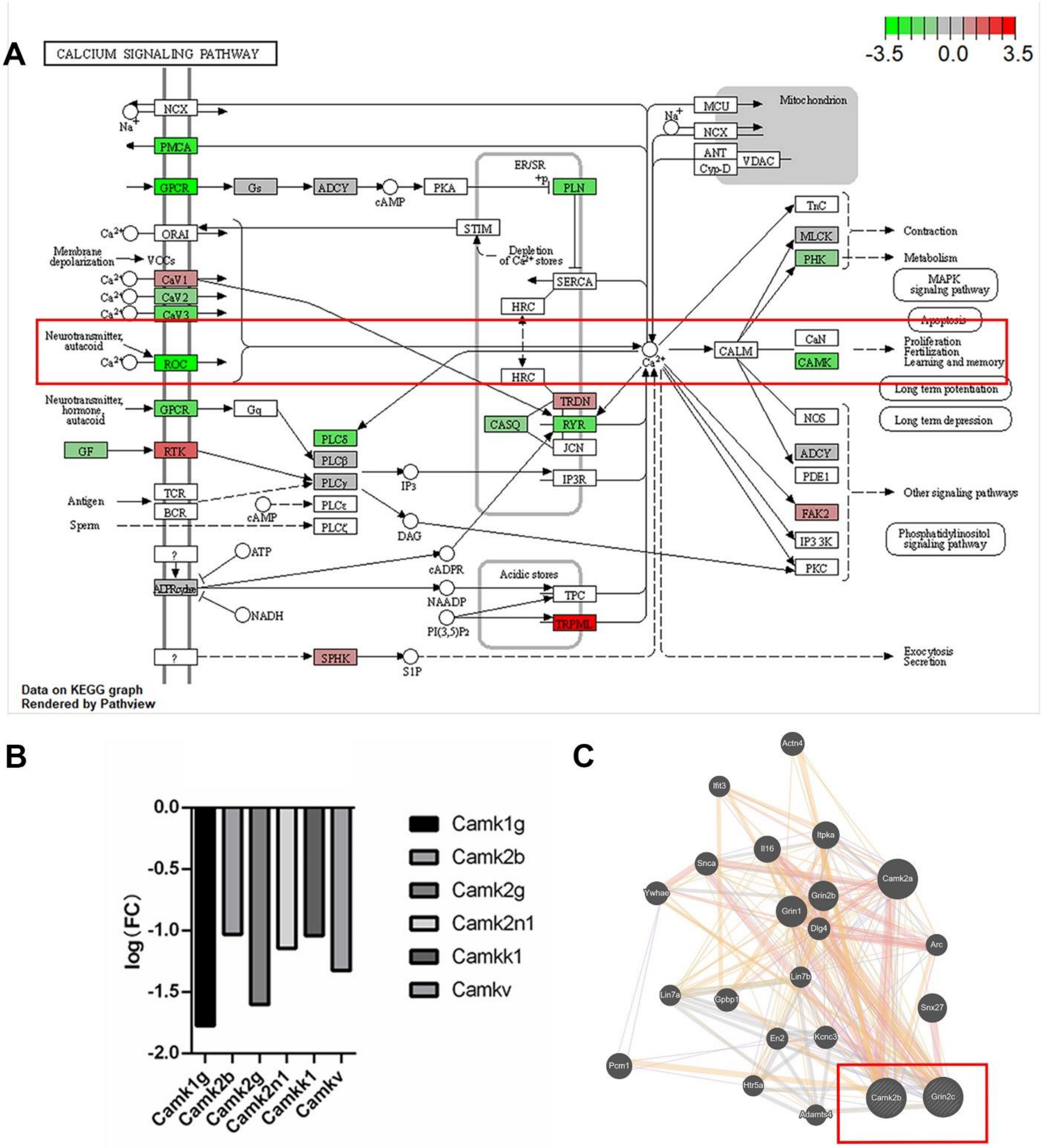
A
CALCIUM SIGNALING PATHWAY
-3.5
0.0
3.5
Mitochondrion
MCU
NCX
ANT
VDAC
Cyp-D
Na+
NCX
PMCA
GPCR
Gs
ADCY
cAMP
PKA
ER/SR
PLN
ORAI
STIM
Membrane depolarization
VOCs
Depletion of Ca2+ stores
CaV1
CaV2
CaV3
SERCA
HRC
Neurotransmitter, autacoid
ROC
CALM
CaN
CAMK
TnC
MLCK
PHK
Contraction
Metabolism
MAPK signaling pathway
Apoptosis
Proliferation
Fertilization
Learning and memory
Long term potentiation
Long term depression
Neurotransmitter, hormone, autacoid
Gq
TRDN
CASQ
RYR
JCN
GF
RTK
PLCδ
PLCβ
PLCγ
PLCε
PLCζ
IP3
IP3R
Antigen
TCR
BCR
Sperm
DAG
NOS
ADCY
PDE1
FAK2
IP3 3K
PKC
Other signaling pathways
Phosphatidylinositol signaling pathway
ATP
NADH
ADPRcyclase
cADPR
NAADP
Acidic stores
TPC
TRPML
PI(3,5)P2
SPHK
S1P
Exocytosis
Secretion
Data on KEGG graph
Rendered by Pathview
B
0.0
-0.5
-1.0
-1.5
-2.0
log（FC）
Camk1g
Camk2b
Camk2g
Camk2n1
Camkk1
Camkv
C
Actn4
Ifit3
Itpka
Il16
Snca
Camk2a
Ywhae
Grin2b
Grin1
Dlg4
Arc
Lin7b
Lin7a
Gpbp1
Snx27
En2
Kcnc3
Pcm1
Htr5a
Camk2b
Grin2c
Adamts4

Fig. S2 The Ca^{2+} signaling pathway of astrocytes is decreased after SCI. **A** This diagram shows the various components of the astrocyte Ca^{2+} signaling pathway after SCI by the Pathview package. Ca^{2+} channel receptors (especially Grin2c) are significantly down-regulated and decreased downstream CaMK can regulate astrocyte proliferation. **B** Fold-changes showing various subtypes of CaMK with down-regulated after SCI, including CaMK1g, CaMK2b, CaMK2g, CaMK2n1, CaMKk1, and CaMKv. **C** The GeneMania website showing that CaMK2b is the potential downstream gene of Grin2c.

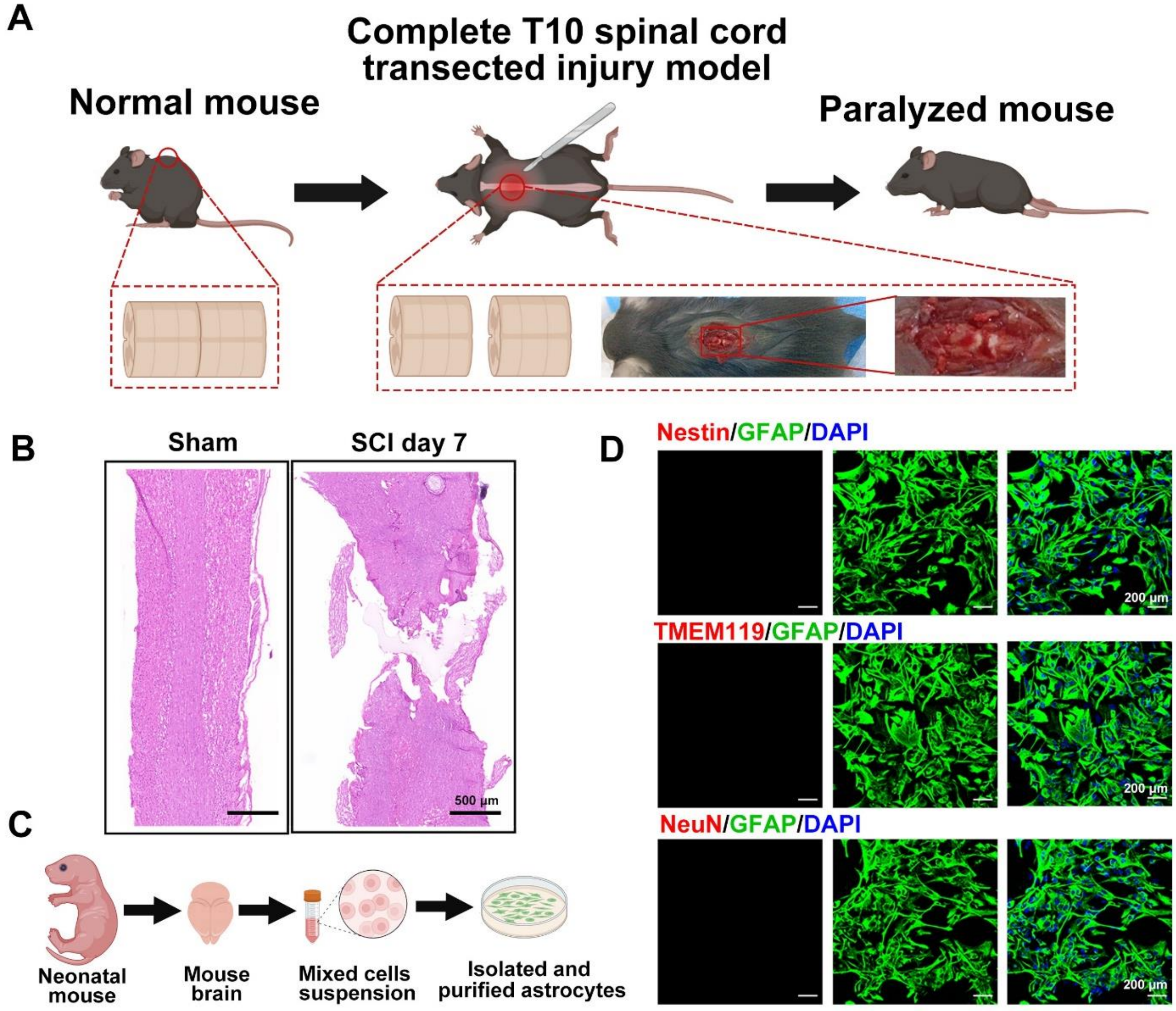

Fig. S3 The construction of a complete T10 spinal cord transected injury model and extraction of primary astrocytes from C57BL/6 mice. **A** Schematic showing complete transection at the 10th thoracic vertebra (T10) SCI model in mice. The diagram shows that the transection of exposed T10 spinal tissue is performed by a scalpel after anesthesia, and mice are paraplegic after SCI. **B** The spinal tissue of mice stained by HE after SCI showing that the spinal tissue is discontinuous and in a state of transverse discontinuity. Scale bars, 500 μm. **C** Schematic of isolated and purified astrocytes from the neonatal mouse brain. **D** Immunofluorescence staining used to determine the purity of primary astrocytes. Nestin/GFAP staining is used to determine whether there are residual neural stem cells in the primary astrocytes, TMEM119/GFAP staining is used to determine whether there are residual microglial cells in the primary astrocytes, and NeuN/GFAP staining is used to determine whether there are residual neurons in the primary astrocytes. Based on the above results, the extracted cells are mainly primary astrocytes, which are used for follow-up experiments. Scale bars, 200 μm.

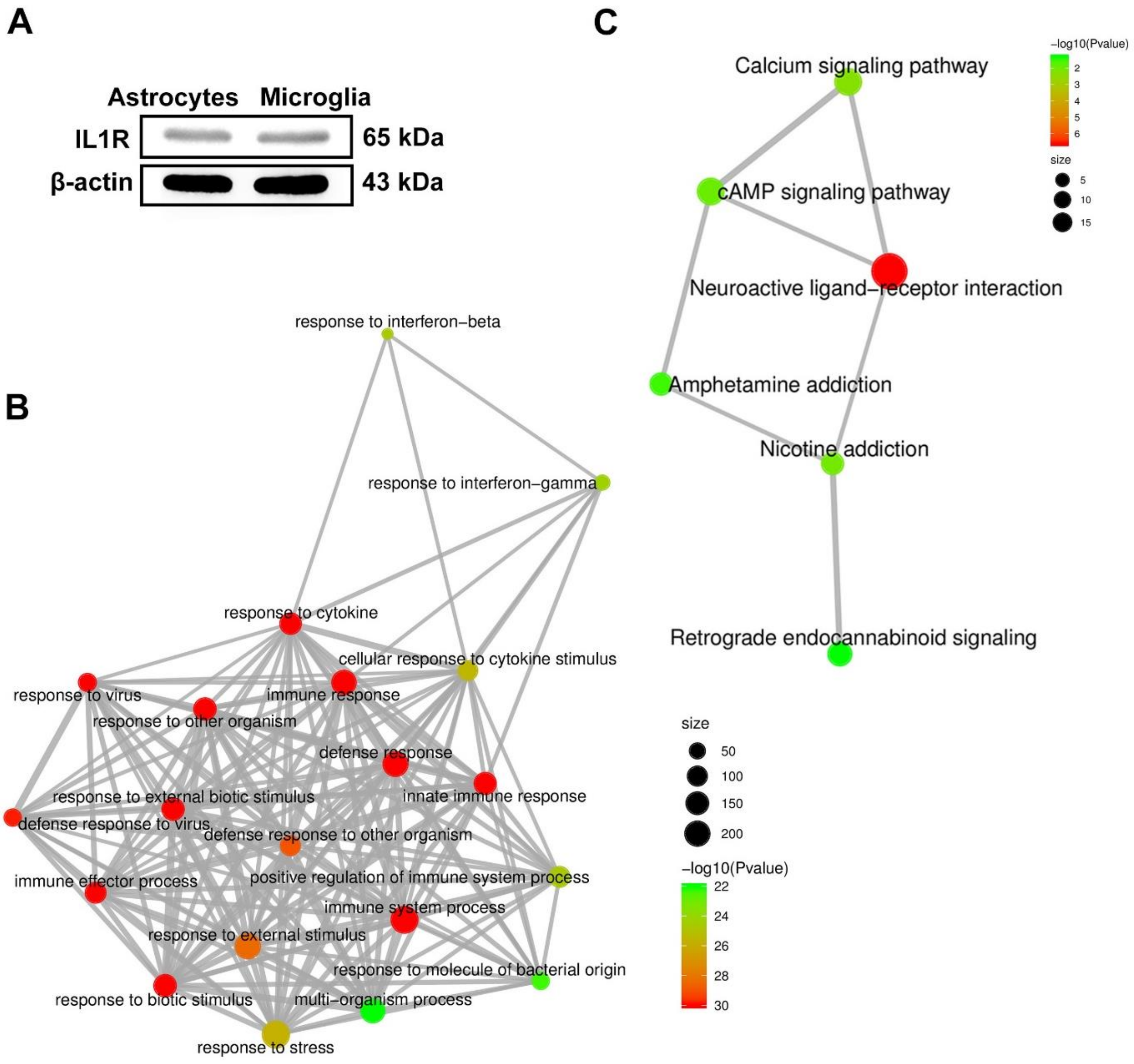

Fig. S4 IL1α can regulate the Ca^{2+} signaling pathway in astrocytes *in vitro*. **A** Representative WB showing that astrocytes have the related receptors of IL1 α**. B** Regulatory interactions of the GO function showing the activation of astrocytes after IL1α stimulation. **C** Regulatory interactions of the KEGG pathway showing that the Ca^{2+} signaling pathway changes after IL1α stimulation. And the results also show that the Ca^{2+} signal pathway is related to the neuroactive ligand-receptor interaction pathway.

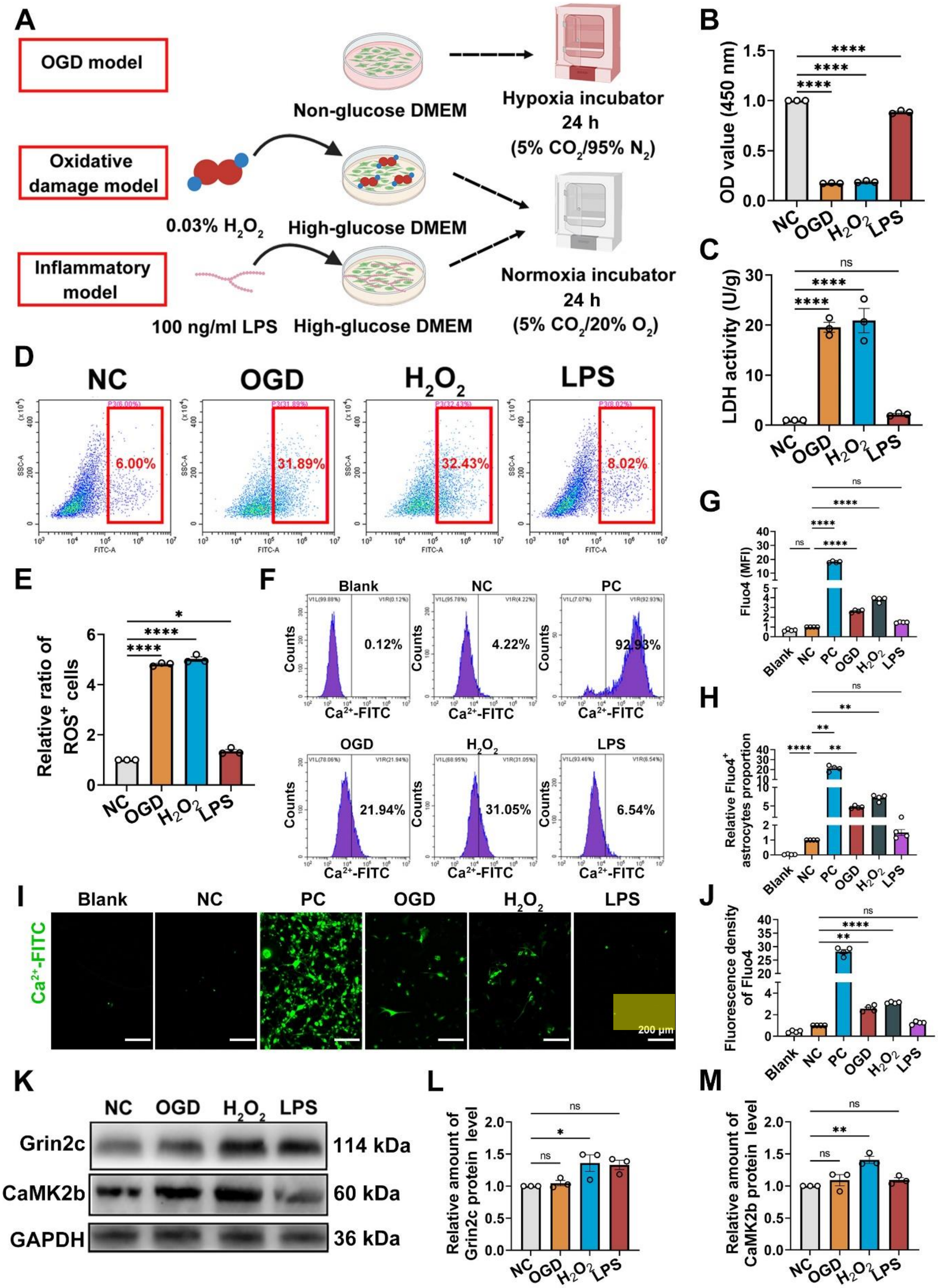
A
OGD model
Non-glucose DMEM
Hypoxia incubator
24 h
(5% CO_2/95% N_2)
Oxidative damage model
0.03% H_2O_2
High-glucose DMEM
Inflammatory model
100 ng/ml LPS
High-glucose DMEM
Normoxia incubator
24 h
(5% CO_2/20% O_2)
B
OD value (450 nm)
NC OGD H_2O_2 LPS
C
LDH activity (U/g)
NC OGD H_2O_2 LPS
D
NC OGD H_2O_2 LPS
6.00% 31.89% 32.43% 8.02%
SSC-A
FITC-A
E
Relative ratio of ROS^+ cells
NC OGD H_2O_2 LPS
F
Blank NC PC
0.12% 4.22% 92.93%
OGD H_2O_2 LPS
21.94% 31.05% 6.54%
Counts
Ca^{2+}-FITC
G
Fluo4 (MFI)
Blank NC PC OGD H_2O_2 LPS
H
Relative $Fluo4^+$ astrocytes proportion
Blank NC PC OGD H_2O_2 LPS
I
Blank NC PC OGD H_2O_2 LPS
Ca^{2+}-FITC
200 μm
J
Fluorescence density of Fluo4
Blank NC PC OGD H_2O_2 LPS
K
NC OGD H_2O_2 LPS
Grin2c 114 kDa
CaMK2b 60 kDa
GAPDH 36 kDa
L
Relative amount of Grin2c protein level
NC OGD H_2O_2 LPS
M
Relative amount of CaMK2b protein level
NC OGD H_2O_2 LPS

Fig. S5 Construction of the damage models of astrocytes *in vitro* to simulate the environment of SCI. **A** Schematic of the *in vitro* oxygen-glucose deprivation (OGD) model, oxidative damage model, and inflammatory model. **B** CCK8 used to assess the viability of astrocytes in different models *in vitro*. Quantitative analysis of the absorbance at 450 nm shows that the OGD model, oxidative damage model, and inflammation model reduce the viability of astrocytes after 24 h. ****P <0.0001, ANOVA with Dunnett's *post hoc* test (n = 3 independent animals). **C** LDH assays for the cytotoxicity of astrocytes in different models. Compared with the NC group, the cytotoxicity of the OGD model and oxidative damage model increase significantly. ****P <0.0001, ANOVA with Dunnett's *post hoc* test (n = 3 independent animals). **D**, **E** ROS assays reflect the degree of oxidative damage of astrocytes in different models. Compared with the NC group, the level of ROS in the OGD model and oxidative damage model increase significantly. ****P <0.0001, *P <0.05, ANOVA with Dunnett's *post hoc* test (n = 3 independent animals). **F**–**H** Flow cytometry and quantification of intracellular Ca^{2+} influx using the fluorescent Ca^{2+} indicator Fluo4. The mean fluorescence intensity (MFI) and relative proportion of Fluo4^{+} cells in astrocytes are significantly increased in the OGD model and oxidative damage model. ****P <0.0001, **P <0.01, ANOVA with Dunnett's *post hoc* test (n = 3 independent animals). **I**, **J** Immunofluorescence staining and quantitative analysis of Fluo4 in astrocytes show a significantly increased level of Ca^{2+} after opening the Ca^{2+} channel. Scale bars, 200 μm, **P <0.0001, *P <0.05, ANOVA with Dunnett's *post hoc* test (n = 3 independent animals). **K** Western blots of Grin2c and CaMK2b. **L**, **M** Quantification of Grin2c and CaMK2b protein levels in the blots show that Grin2c/CaMK2b levels in the oxidative damage model group are significantly increased (Grin2c H_2O_2 *vs* NC, *P <0.05; CaMK2b H_2O_2 *vs* NC *P <0.05). ANOVA with Dunnett's *post hoc* test (n = 3 independent animals).

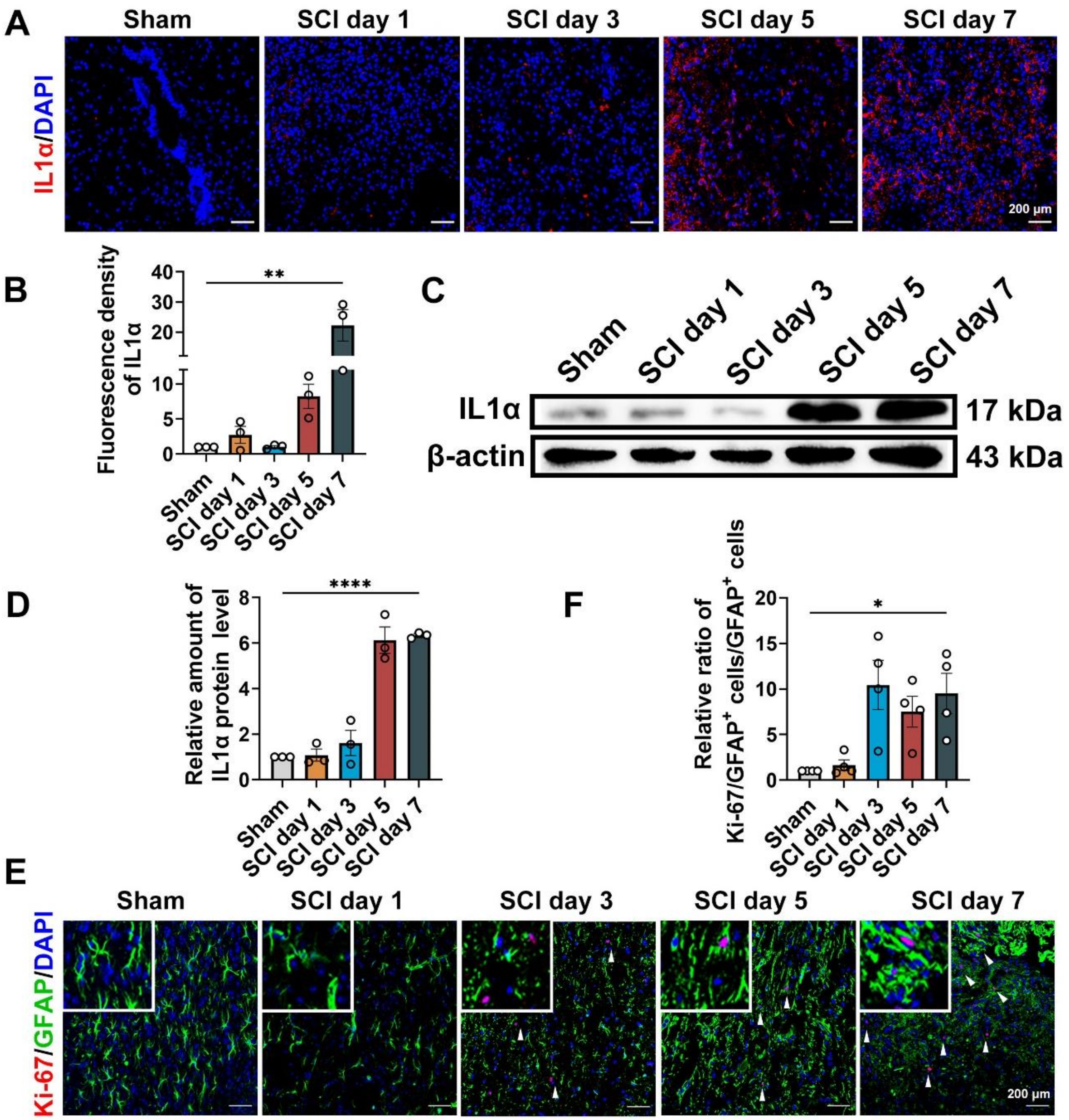

Fig. S6 Astrocytes proliferate and IL1α levels are upregulated around the damaged region in SCI mice. **A** Immunofluorescence staining of IL1α in the damaged area on different days after SCI. Scale bars, 200 μm. **B** IL1α fluorescence intensity and the level of IL1α are significantly increased on the 7th day after SCI. ****P <0.0001, ANOVA with Dunnett's *post hoc* test (n = 3 independent animals). **C** WB assays of

the protein level of IL1α around the damaged region after SCI. **D** The protein level of IL1α increases significantly on the 7th day after SCI. ****P <0.0001, ANOVA with Dunnett's *post hoc* test, n = 3 independent animals. **E** Immunofluorescence staining of Ki-67/GFAP around the damaged region on different days following SCI. Scale bars, 200 μm. **F** Ratios of Ki-67$^+$/GFAP$^+$ cells show the proliferation of astrocytes is increased after SCI (SCI day 7 *vs* Sham, ***P <0.001), ANOVA with Dunnett's post hoc test (n = 3 independent animals).

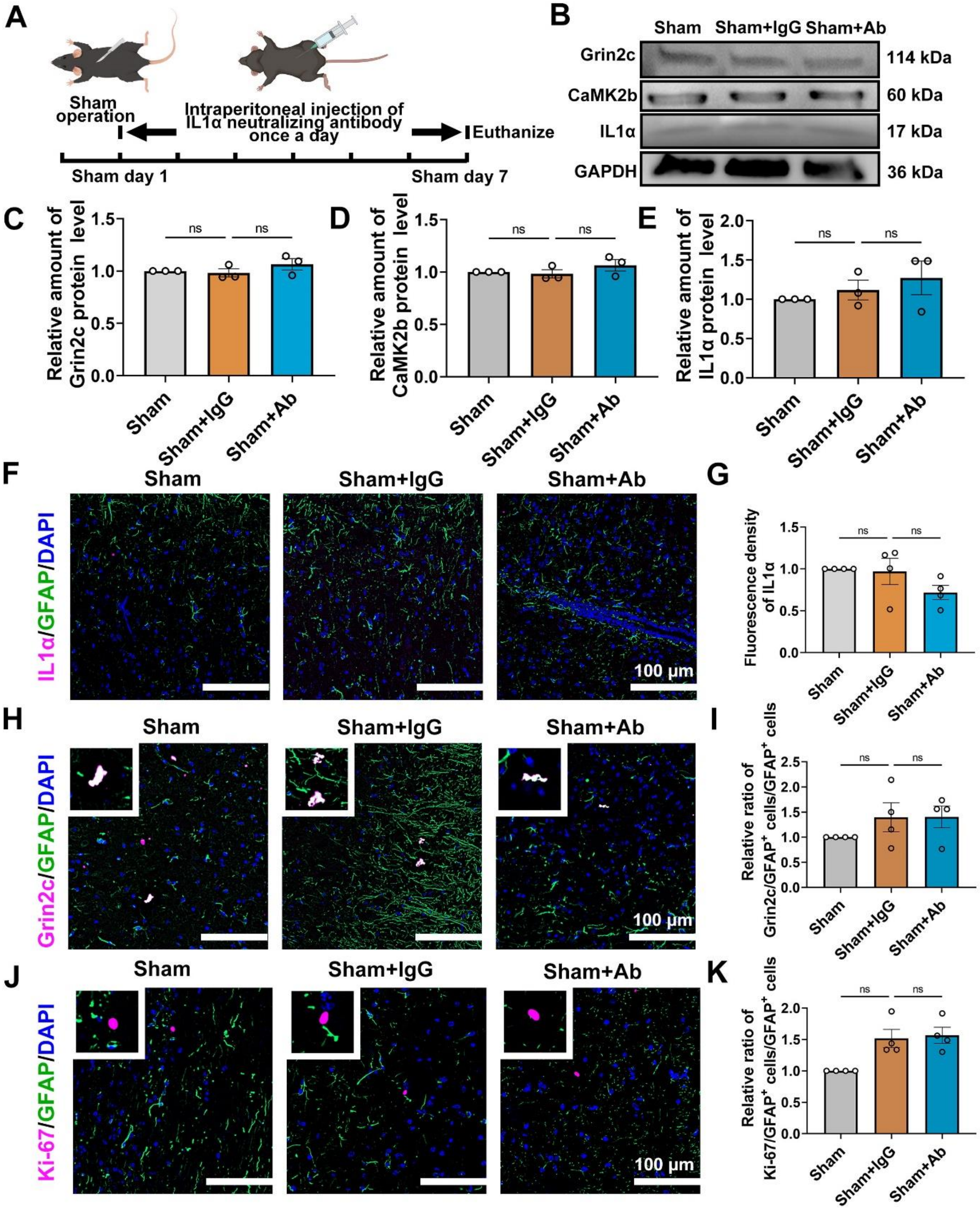

A
Sham operation
Intraperitoneal injection of IL1α neutralizing antibody once a day
Euthanize
Sham day 1
Sham day 7
B
Sham
Sham+IgG
Sham+Ab
Grin2c
CaMK2b
IL1α
GAPDH
114 kDa
60 kDa
17 kDa
36 kDa
C
Relative amount of Grin2c protein level
D
Relative amount of CaMK2b protein level
E
Relative amount of IL1α protein level
ns
F
IL1α/GFAP/DAPI
100 μm
G
Fluorescence density of IL1α
H
Grin2c/GFAP/DAPI
I
Relative ratio of Grin2c/GFAP+ cells/GFAP+ cells
J
Ki-67/GFAP/DAPI
K
Relative ratio of Ki-67/GFAP+ cells/GFAP+ cells

Fig. **S7** Blockade of IL1α using neutralizing antibodies in Sham mice. **A** Schematic of intraperitoneal neutralizing antibody injection in sham mice. **B** The protein levels of Grin2c, IL1α, and CaMK2b in the spinal cord determined by WB assays after injection of neutralized antibody. **C**–**E** IL1α, Grin2c, and CaMK2b protein levels in WB assays. The level of IL1α does not cause a notable change after injection of IL1α neutralizing antibody. Compared with the Sham+IgG group (injection control group), there is no significant change in Grin2c and CaMK2b in the Sham+Ab group. ns, no significant difference, ANOVA with Dunnett's *post hoc* test (n = 3 independent animals). **F**, **G** Immunofluorescence staining and quantitative analysis of IL1α (red) in the Sham+Ab group. There no significant change in the level of IL1α in the spinal environment after injection of neutralizing antibody. Scale bars, 100 μm; ns, no significant difference, ANOVA with Dunnett's *post hoc* test (n = 4 independent animals). **H** Grin2c/GFAP immunofluorescence staining of spinal sections after injection of neutralizing antibody. Scale bars,100 μm. **I** Ratios of $Grin2c^{+}/GFAP^{+}$ cells showing that the Grin2c of astrocytes is invariant after blockade of IL1α in sham mice. ns, no significant difference, ANOVA with Dunnett's *post hoc* test (n = 4 independent animals). **J** Immunofluorescence staining of Ki-67/GFAP following the blockade of IL1α. Scale bars, 100 μm. **K** Ratios of $Ki\text{-}67^{+}/GFAP^{+}$ cell showing that neutralizing IL1α does not effectively influence the proliferation of astrocytes in sham mice. ns, no significant difference, ANOVA with Dunnett's *post hoc* test (n = 4 independent animals).

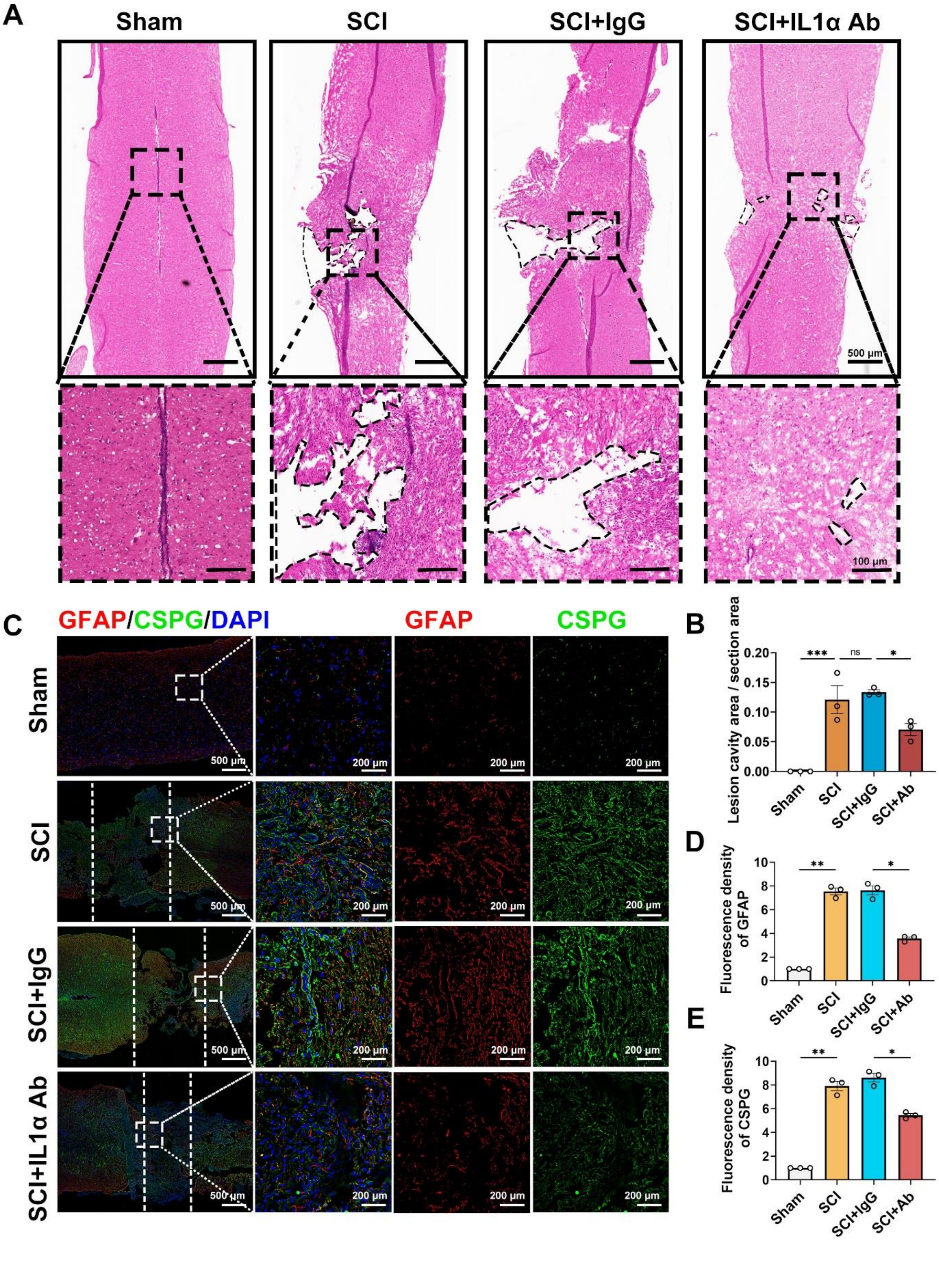
A
Sham
SCI
SCI+IgG
SCI+IL1α Ab
500 μm
100 μm
C
GFAP/CSPG/DAPI
GFAP
CSPG
Sham
SCI
SCI+IgG
SCI+IL1α Ab
500 μm
200 μm
B
Lesion cavity area / section area
0.00
0.05
0.10
0.15
0.20
***
ns
*
Sham
SCI
SCI+IgG
SCI+Ab
D
Fluorescence density of GFAP
0
2
4
6
8
10
**
*
E
Fluorescence density of CSPG

Fig. S8 Blockade of IL1α using neutralizing antibodies reduces the lesion scar and lesion volume in SCI mice. **A**, **B** The spinal tissue of mice stained by HE after SCI showing the lesion on the 7th day after surgery. Compared with the SCI+IgG group, the lesion volume is decreased after injection of IL1α neutralizing antibodies. Scale bars, 500 μm and 100 μm. ***P <0.001, *P <0.05, ANOVA with Dunnett's *post hoc* test (n = 3 independent animals). **C** Immunofluorescence staining of CSPG (chondroitin sulfate proteoglycans) (green)/GFAP (red) in the damaged area after SCI. Both stains represent scar tissue to a certain extent; CSPG is one main cellular component of scars, and astrocytes (GFAP$^+$ cells) are involved in scar formation. Scale bars, 500 μm and 200 μm. **D**–**F** Immunofluorescence staining shows that CSPG and GFAP levels in the damaged area were significantly decreased after the blockade of IL1α in SCI mice. **P <0.01, *P <0.05, ANOVA with Dunnett's *post hoc* test (n = 3 independent animals).

Table S1 Primer sequences of genes for qRT-PCR

**Gene Names**	**Forward primer (5'-3')**	**Reverse primer (5'-3')**	**Length (bp)**
grin2c	GGGATCTGCCATAACGAGAAG	GCACTGAGTGTCGAAGTTTCCA	157
camk2b	CGTTTCACCGACGAGTACCAT	GGCAGATCCGAGCTTCTCT	172
actb	GGCTGTATTCCCCTCCATCG	CCAGTTGGTAACAATGCCATGT	154

Sequences of Grin2c-OE and Grin2c-SiRNA

	**mGrin2c-SiRNA sense**	**mGrin2c-SiRNA antisense**
**Grin2c-SiRNA**	CCAGGCCAAUGUGCUGAAGAUTT	AUCUUCAGCACAUUGGCCUGGTT

**Grin2c-OE**	ATGGGTGGAGCCCTGGGGCCCGCCCTGCTTCTCACTTCACTCCTTGGTG<br>CTTGGGCAGGGCTGGGCGCAGGGCAGGGAGAACAGGCCGTGACCGTG<br>GCGGTGGTGTTTGGCAGCTCTGGGCCACTGCAGGCCCAGGCCCGGACT<br>CGTCTCACCCCGCAGAACTTCCTGGACTTGCCTCTGGAGATCCAGCCAC<br>TCACCATCGGGGTCAACAATACCAACCCCAGCAGCATCCTCACCCAAA<br>TCTGTGGGCTCCTGGGTGCCGCCCGAGTCCACGGCATCGTCTTTGAGGA<br>CAACGTGGACACTGAGGCCGTGGCTCAGCTGCTGGATTTCGTCTCCTCT<br>CAGACCCACGTGCCCATCCTCAGCATCAGTGGAGGTTCTGCTGTGGTCC<br>TCACCCCCAAGGAGCCAGGCTCCGCCTTTCTACAGCTGGGCGTGTCCCT<br>GGAGCAGCAGCTGCAGGTGCTGTTCAAGGTGCTGGAGGAATACGACTG<br>GAGCGCGTTCGCTGTCATCACCAGCCTGCACCCGGGCCACGCGCTCTTC<br>CTCGAGGGCGTGCGCGCCGTCGCCGACGCCAGCTACCTGAGCTGGCGG<br>CTGCTGGACGTGCTCACGCTGGAGCTGGGCCCCGGTGGGCCGCGAGCG<br>CGCACTCAGCGCTTACTGCGCCAGGTCGACGCCCCGGTGCTGGTGGCCT<br>ACTGCTCCCGTGAAGAGGCGGAGGTGCTCTTCGCGGAGGCTGCACAGG<br>CTGGCTTGGTGGGACCCGGTCACGTGTGGTTAGTACCTAATCTGGCGCT<br>GGGAAGCACCGACGCTCCCCCTGCAGCCTTCCCAGTGGGCCTCATCAG<br>TGTGGTCACCGAGAGTTGGCGCCTTAGCCTACGCCAGAAAGTCCGCGA<br>CGGTGTAGCCATTCTGGCCCTCGGTGCCCACAGCTACCGACGCCAGTAC<br>GGTACCCTTCCAGCCCCGGCTGGAGACTGCCGAAGCCACCCAGGACCC<br>GTCAGCCCTGCCAGGGAGGCTTTCTACAGGCATCTGCTGAATGTCACCT<br>GGGAAGGCCGAGACTTCTCTTTTAGCCCTGGTGGGTACCTGGTCCAGCC<br>CACAATGGTTGTGATCGCTCTCAACCGGCATCGCCTCTGGGAGATGGTG

GGACGGTGGGATCATGGGGTCCTGTACATGAAGTATCCAGTATGGCCT

CGCTACAGCACTTCTCTGCAGCCTGTGGTGGACAGCCGGCACCTGACA

GTGGCCACACTGGAAGAAAGGCCTTTTGTCATTGTGGAGAGCCCTGAC

CCTGGCACAGGTGGCTGTGTTCCCAACACTGTGCCCTGCCGTAGACAG

AGCAACCACACCTTCAGCAGCGGGGATATAACCCCCTACACCAAGCTC

TGTTGTAAGGGCTTCTGCATCGACATCCTCAAGAAGCTGGCCAAGGTG

GTCAAGTTCTCCTACGACTTGTACCTGGTGACCAACGGCAAGCACGGC

AAGAGGGTTCGTGGTGTGTGGAATGGTATGATCGGTGAGGTATACTAC

AAGCGGGCAGACATGGCCATCGGCTCCCTCACCATCAATGAAGAGCGC

TCAGAGATTATAGACTTCTCTGTGCCTTTTGTGGAGACCGGCATCAGTG

TGATGGTGGCAAGGAGCAACGGCACCGTCTCCCCCTCGGCTTTTCTGGA

GCCCTACAGCCCTGCCGTGTGGGTGATGATGTTTGTAATGTGCCTCACG

GTGGTTGCCATCACTGTCTTCATGTTCGAGTATTTCAGCCCTGTCAGCT

ACAACCAGAATCTCACCAAGGGCAAGAAGTCAGGTGGACCATCCTTCA

CCATTGGCAAGTCCGTGTGGTTGCTGTGGGCACTGGTCTTCAACAACTC

TGTTCCCATCGAGAACCCCCGGGGCACCACCAGCAAGATCATGGTCCT

GGTGTGGGCCTTCTTCGCTGTCATCTTCCTCGCTAGCTACACGGCCAAT

CTGGCAGCCTTCATGATCCAGGAACAATACATCGACACTGTGTCGGGC

CTTAGTGACAAGAAGTTTCAGCGGCCTCAAGACCAATACCCACCCTTCC

GTTTTGGCACGGTACCTAATGGCAGCACAGAGAGGAACATTCGTAGCA

ACTATCGTGACATGCACACTCACATGGTCAAGTTCAACCAGCGCTCGGT

GGAGGATGCTCTCACAAGCCTGAAGATGGGGAAGCTGGACGCCTTCAT

CTATGATGCCGCCGTCCTCAACTACATGGCGGGCAAGGACGAAGGCTG

CAAGCTGGTCACCATTGGGTCTGGCAAAGTCTTTGCCACCACTGGCTAT
GGCATTGCCATGCAGAAAGACTCCCACTGGAAGCGGGCCATAGACCTG
GCGCTCCTGCAGTTCCTGGGGGATGGGGAGACACAGAAGTTGGAGACA
GTGTGGCTCTCAGGGATCTGCCATAACGAGAAGAACGAGGTGATGAGC
AGCAAGCTGGACATTGACAACATGGCGGGCGTCTTCTACATGCTGTTG
GTGGCCATGGGGCTGGCCCTTCTGGTCTTTGCCTGGGAGCACCTGGTCT
ACTGGAAACTTCGACACTCAGTGCCCAGCTCATCCCAGCTGGACTTCCT
GCTGGCTTTCAGCAGGGGCATCTACAGCTGCTTCAACGGGGTACAGAG
CCTTCCGAGCCCTGCGCGGCCGCCCAGCCCGGACCTTACAGCAGGCTC
AGCCCAGGCCAATGTGCTGAAGATGCTGCAGGCGGCTCGAGACATGGT
GAGCACAGCGGACGTGAGCGGCTCTTTGGACCGCGCCACTCGTACCAT
CGAGAACTGGGGCAACAATCGCCGCGCGCCTGCTCCCACCACCTCCGG
CCCGCGGTCATGCACCCCGGGTCCTCCGGGACAACCGAGTCCCAGCGG
CTGGCGGCCTCCCGGTGGGGGCCGCACCCCGCTAGCGCGCCGGGCCCC
GCAGCCTCCCGCTCGCCCCGCGACCTGCGCAGGGTCGCCTCAGCCCGA
CGTGTCCCGAGCATCCTGCAGGCACGCTTGGGATGCGCGGTGGCCAGT
GCGAGTCGGGCATCAGGGATCGCACCTCTCGGCCTCCGAGCGGCGCGC
GCTCCCGGAGCGCTCCCTGTTGCACGCGCACTGCCACTACAGTTCCTTC
CCTCGAGCAGAGAGGTCAGGGCGCCCATTTCTCCCGCTATTCCCGGAG
CCCCCGGAGCCCGACGACCTGCCGCTGCTCGGGCCGGAACAGCTGGCT
CGGCGGGAGGCTCTGCTGCGCGCGGCCTGGGCCAGGGGCCCGCGCCCT
CGGCACGCTTCCCTGCCCAGCTCCGTGGCAGAAGCCTTCACTCGATCCA
ACCCTCTGCCTGCCAGGTGTACCGGTCACGCCTGCGCTTGCCCATGTCC

CCAAAGCCGGCCATCCTGCCGGCACGTGGCTCAAACACAGTCGTTGCG

GCTGCCATCCTACCGGGAGGCCTGTGTGGAGGGCGTGCCAGCAGGGGT

GGCCGCCACCTGGCAGCCCAGACAGCATGTCTGCCTGCACACCCATAC

CCACCTGCCGTTCTGCTGGGGGACTGTCTGCCGTCACCCTCCACCCTGT

TCCAGCCACAGTCCCTGGCTCATTGGAACTTGGGAGCCTCCATCACACA

GAGGCAGGACCCTGGGGCTAGGTACAGGCTACAGGGACAGTGGGGTG

CTAGAAGAGGTCAGCAGGGAAGCTTGTGGGACACAAGGGTTTCCAAGG

TCCTGCACCTGGAGGCGGATCTCCAGCCTGGAATCAGAAGTGTGA
